# Evidence for Common Horizontal Transmission of *Wolbachia* among Ants and Ant Crickets: Kleptoparasitism Added to the List

**DOI:** 10.3390/microorganisms8060805

**Published:** 2020-05-27

**Authors:** Shu-Ping Tseng, Po-Wei Hsu, Chih-Chi Lee, James K. Wetterer, Sylvain Hugel, Li-Hsin Wu, Chow-Yang Lee, Tsuyoshi Yoshimura, Chin-Cheng Scotty Yang

**Affiliations:** 1 Research Institute for Sustainable Humanosphere, Kyoto University, Kyoto 611-0011, Japan; tseng.shuping.65x@st.kyoto-u.ac.jp (S.-P.T.); briangd2s@gmail.com (P.-W.H.); lee.chihchi.54m@st.kyoto-u.ac.jp (C.-C.L.); tsuyoshi@rish.kyoto-u.ac.jp (T.Y.); 2 Laboratory of Insect Ecology, Graduate School of Agriculture, Kyoto University, Kyoto 606-8502, Japan; 3 Wilkes Honors College, Florida Atlantic University, 5353 Parkside Drive, Jupiter, FL 33458, USA; wetterer@fau.edu; 4 Institut des Neurosciences Cellulaires et Intégratives, UPR 3212 CNRS-Université de Strasbourg, 67000 Strasbourg, France; hugels@inci-cnrs.unistra.fr; 5 Department of Plant Medicine, National Pintung University of Science and Technology, Pintung 91201, Taiwan; lihsinwuu@gmail.com; 6 Department of Entomology, University of California, 900 University Avenue, Riverside, CA 92521, USA; chowyang.lee@ucr.edu; 7 Department of Entomology, Virginia Polytechnic Institute and State University, Blacksburg, VA 24061, USA; 8 Department of Entomology, National Chung Hsing University, Taichung 402204, Taiwan

**Keywords:** generalist, horizontal transmission, kleptoparasitism, specialist, *Wolbachia*

## Abstract

While *Wolbachia*, an intracellular bacterial symbiont, is primarily transmitted maternally in arthropods, horizontal transmission between species has been commonly documented. We examined kleptoparasitism as a potential mechanism for *Wolbachia* horizontal transmission, using ant crickets and their host ants as the model system. We compared prevalence and diversity of *Wolbachia* across multiple ant cricket species with different degrees of host specificity/integration level. Our analyses revealed at least three cases of inter-ordinal *Wolbachia* transfer among ant and ant crickets, and also showed that ant cricket species with high host-integration and host-specificity tend to harbor a higher *Wolbachia* prevalence and diversity than other types of ant crickets. This study provides empirical evidence that distribution of *Wolbachia* across ant crickets is largely attributable to horizontal transmission, but also elucidates the role of intimate ecological association in successful *Wolbachia* horizontal transmission.

## 1. Introduction

*Wolbachia* are widespread, maternally-transmitted, intracellular bacteria, present in approximately half of all arthropod species [1,2]. Discordant phylogenies between *Wolbachia* and their hosts suggest that transmission of *Wolbachia* also includes horizontal transmission between species [3,4,5,6], which can be analogous to an epidemiological process mediated by the ability of a pathogen to invade and maintain in novel host populations [7]. *Wolbachia* must pass three main filters before successfully colonizing a new host species [8]. First, *Wolbachia* must come into physical contact with the potential host (encounter filter), then evade the host’s immune system and replicate in the new host (compatibility filter). Whether *Wolbachia* can reach a certain infection threshold to ensure its persistence in the population represents the third filter (invasion filter). The community composition may affect filter stringency and thus shapes the epidemiological patterns of *Wolbachia* in a community. Communities composed of generalist or specialist species will affect both encounter and compatibility filters [9,10]. For example, the intimate interspecific interactions by specialists are predicted to favor *Wolbachia* transmission. However, these interactions may also restrict the transmission sources to a few species [9].

It has been considered that intimate tissue-level interactions between donor and recipient species such as host-parasitoid, host-parasite, or prey-predator interactions are required for interspecific transmission of *Wolbachia* [11,12,13,14,15]. Nevertheless, some studies have shown that the presence of identical *Wolbachia* strains among species that do not share parasitoids and/or predators but habitats, suggesting other mechanisms may have been involved in *Wolbachia* horizontal transmission [10,16,17,18,19]. Previous studies have reported horizontal transfer of *Wolbachia* between ant hosts and their parasitic ants most likely through intimate contacts [17,18,19], however, whether such a mechanism holds true for distantly related species (e.g., inter-ordinal transfers) remains unknown. Ant nests are often utilized by other myrmecophiles [20], offering an excellent opportunity to test if *Wolbachia* horizontal transmission remains feasible among distantly related species. Ant crickets (Orthoptera: Myrmecophilidae) are generally considered as kleptoparasites (i.e., parasitism by theft, as a form of resource acquisition where one animal takes resources from another), and represent an intriguing group of myrmecophilous taxa that display differential host specificity and integration levels, rendering them suitable examining for *Wolbachia* horizontal transmission at the inter-ordinal level. In the present study, we conducted an extensive *Wolbachia* survey in kleptoparasitic ant crickets and their corresponding ant hosts and asked whether inter-ordinal transfer of *Wolbachia* occurs frequently through kleptoparasitism. The potential interplays between ant crickets of different host specificity/integration levels and *Wolbachia* infection patterns are also discussed.

## 2. Materials and Methods

### 2.1. Insect Collection, DNA Extraction and PCR Conditions

Ant crickets can be generally classified as three groups according to their host specificity and integration levels: integrated host-specialist, non-integrated host-specialist and host-generalist ant crickets [21,22] (see Tables S1 and S2 for a detailed definition). Integrated host-specialist ant crickets possess low survivorship in the absence of ant hosts, engaging in intimate behavioral interactions with their ant hosts, such as trophallaxis [21]. Despite differences in host specificity, both non-integrated host-specialist and host-generalist ant crickets feed independently, often escape ant attack by swift movements and are capable of surviving without ants [21,22]. Seven ant cricket species and their ant hosts from the same colonies were collected and used in this study, including two integrated host-specialist species *Myrmecophilus americanus* (*n* = 40) and *M*. *albicinctus* (*n* = 38), three non-integrated host-specialist species *M*. *dubius* (*n* = 23), *M*. *hebardi* (*n* = 26) and *M*. *antilucanus* (*n* = 26), and two host-generalist species *M*. *quadrispina* (*n* = 31) and *Myrmophilellus pilipes* (*n* = 23). Most of the ant cricket species and their ant hosts were collected from Asia (Table S3). 

*Myrmecophilus albicinctus*, *M*. *antilucanus*, *M*. *dubius* and *M*. *hebardi* are host-specialists associated with the yellow crazy ant, *A. gracilipes*, whereas *M*. *americanus* is exclusively associated with the longhorn crazy ant, *Paratrechina longicornis* [23,24]. The two host-generalist species have been reported in nests of more than ten ant species each [21,23,25], and our host-generalist samples were collected from ant colonies of six ant species (Tables S2 and S3).

Genomic DNA was extracted from the legs of ant crickets and their ant hosts using the Gentra Puregene cell and tissue kit (Qiagen, Frederick, MD, USA). To detect *Wolbachia* infection, polymerase chain reaction (PCR) was employed to amplify the partial *Wolbachia* surface protein gene (*wsp*) (primers are listed in Table S4 and PCR conditions follow [26]), with inclusion of proper positive control and blank (ddH_2_O). When necessary, to distinguish multiple sequences from individuals with multiple *Wolbachia* infections, the amplified products of the *wsp* gene were cloned using the TOPO TA cloning kit (Invitrogen, Carlsbad, CA, USA). Twenty colonies were selected from each PCR reaction and sequenced. To further confirm the infection status of individuals with multiple infections, six additional specific primer sets were designed based on the sequencing results of the cloning experiment (Table S4) and each amplicon was also sequenced. *Wolbachia* strains were characterized using the multi-locus sequence typing (MLST) system [3]. Five MLST loci (*hcpA*, *ftsZ*, *gatB*, *coxA* and *fbpA*; 2565 bp in total) were amplified and sequenced following the protocols described on the PubMLST (https://pubmlst.org/Wolbachia/info/protocols.shtml). Each *Wolbachia* strain was assigned a sequence type (ST; a unique series of alleles) that defines a combination of five alleles (i.e., allelic profile) at MLST loci. Strains with alleles identical at the five MLST loci are assigned the same ST. For individuals with multiple *Wolbachia* infections, we relied on next-generation sequencing technologies for identification of MLST alleles and assigned MLST alleles to strains using available allelic profile information for reference (see Supplementary Materials Figure S1 for more details). Obtained *Wolbachia* sequences were deposited in GenBank and the *Wolbachia* MLST database (see Table 1 for GenBank accession numbers and MLST ids).

### 2.2. Sequence Alignment and Phylogenetic Analyses

The phylogenetic status of identified *Wolbachia* strains and co-phylogenetic patterns of *Wolbachia* and ant crickets were examined based on three datasets: (1) concatenated *wsp* and MLST sequences (2) MLST sequences and (3) partial mtDNA *cytb* sequences of the crickets. The nucleotide sequences of *wsp*, MLST and *cytb* sequences were aligned separately on the GUIDANCE2 Server [27] based on codons using the MAFFT algorithm [28]. Ambiguous alignments (e.g., those with the confidence score below 0.7) were excluded, resulting in a removal of 12.4% of columns of *wsp* gene.

We inferred phylogenetic relationships of *Wolbachia* strains based on concatenated sequences of *wsp* and MLST using the maximum-likelihood (ML) method with RAxML Blackbox web-servers [29] implementing the optimal substitution model and partitions estimated in PartitionFinder version 2.1.1 [30]. Phylogenies of *Wolbachia* MLST were inferred using both the maximum likelihood (ML) and the Bayesian method with the RAxML Blackbox and ClonalFrame 1.1 [31]. In addition to *Wolbachia* sequence types (STs) detected from ant crickets and ant hosts generated in this study, several other STs (the most similar STs found in the MLST database, selected representative STs from each *Wolbachia* supergroup and Formicidae STs available in the MLST database) were included in the MLST phylogenetic analysis. ML phylogeny was inferred using the RAxML Blackbox implementing the optimal substitution model and partitions estimated in PartitionFinder version 2.1.1 [30]. Bayesian analyses were conducted using ClonalFrame 1.1 [31]. Two independent runs were performed with 1,000,000 generations each, a sampling frequency of 1000 and a burn-in of 50%. A 50% majority rule consensus tree was built from combined data from the two independent runs. We inferred phylogenetic relationships of ant crickets based on the partial mtDNA *cytb* gene using the ML method. Sequences of partial mtDNA *cytb* gene for ant crickets were obtained from a previous study [23] (GenBank accession number: MN064914–MN065077), and inference of ML phylogeny followed the first two datasets.

## 3. Results

We identified ten and six *Wolbachia* strains from the studied ant crickets and ants, respectively (Table 1). Nine of the ten *Wolbachia* strains from ant crickets were fully typed by MLST and assigned to a sequence type (ST) (Table 1). *w*Msp1–*w*Msp8 and *wMame1* are represented by seven unique MLSTs belonging to either *Wolbachia* supergroup A or F (Table 1). We excluded *w*Mame2 from the MLST analysis because the *wsp* sequence of *w*Mame2 differed from all known *Wolbachia* strains in the PubMLST database, and individual crickets bearing the *w*Mame2 strain were always found to be infected with other closely related strains. *Wolbachia w*LonA, *w*LonF, *w*Agra and *w*CamA1–*w*CamA3 from ants are represented by five unique STs (Table 1). Note the allelic profile of strains *w*Mame2 and *w*CamA1–*w*CamA3 were assigned based on available allelic profile information as reference, assuming no recombination among strains (see supplementary materials for more details).

**Figure 1:**
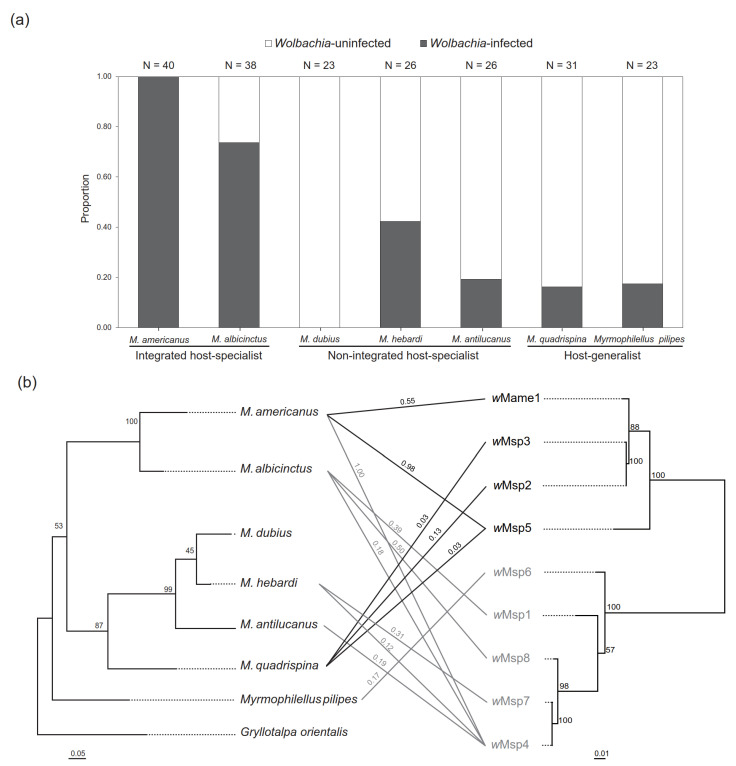
(**a**) *Wolbachia* infection rates of ant crickets; (**b**) Maximum Likelihood (ML) phylogeny of ant crickets (left) and their corresponding *Wolbachia* strains (right) based on *cytb* sequences and concatenated sequences of *wsp* and MLST, respectively. Numbers at nodes indicate bootstrap support values (100 replicates). Ant cricket-*Wolbachia* associations are indicated by lines (black: supergroup A; gray: supergroup F), and the number above the line indicates the infection rate of each *Wolbachia* strain. *Wolbachia* strain *w*Mame2 was excluded from the phylogenetic analysis due to the lack of reliable MLST data.

*Wolbachia* infection frequency varied across ant cricket species, with the highest frequency (100%) observed in *M. americanus* and the lowest (0%) observed in *M. dubius* (Figure 1a). Most ant crickets were infected with supergroup A *Wolbachia*, and *w*Msp4 was among the most widespread strains, which was shared among four species (Figure 1b). Supergroup F *Wolbachia* was found in two phylogenetically distant species, *M. americanus* and *M. quadrispina* (Figure 1b). Comparisons of phylogenetic trees of *Wolbachia* and their cricket hosts indicated no evidence of cricket-*Wolbachia* co-divergence (Figure 1b). *Myrmecophilus americanus* harbored the highest *Wolbachia* frequency and diversity, with most infected individuals harboring more than two *Wolbachia* strains (triple infection: 43%; quadruple infection: 55%), while single or double infections were common in other species (Table S3).

**Figure 2:**
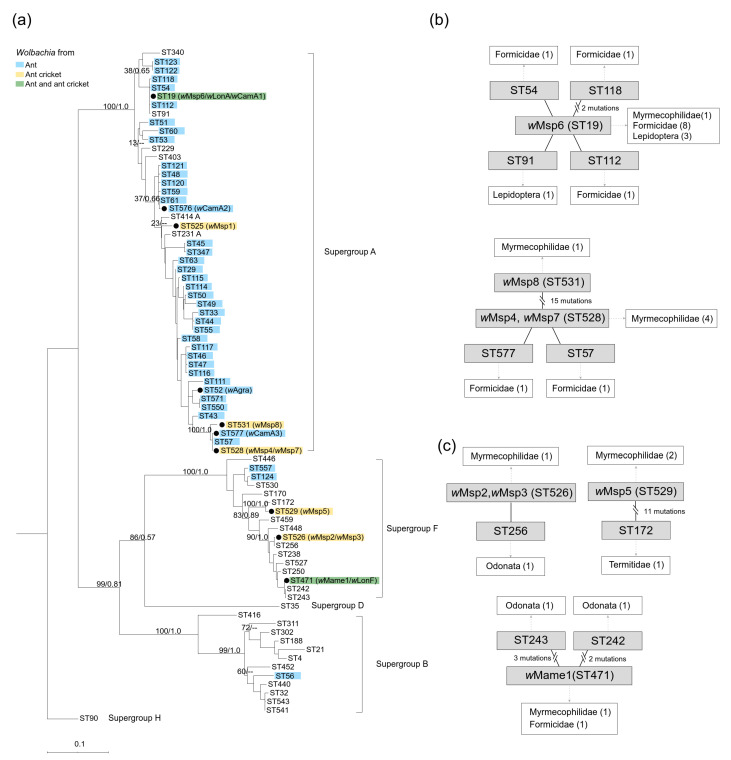
(**a**) Maximum Likelihood (ML) phylogeny of *Wolbachia* based on MLST data, with ML bootstrap values (left) and Bayesian posterior probability (right) given. Note that both Bayesian inference and ML yielded a highly similar topology. Black circles denote the *Wolbachia* STs identified in the present study. *Wolbachia* from ants, ant crickets and *Wolbachia* shared between ants and ant crickets are colored blue, yellow and green, respectively. Relationships among supergroup A *Wolbachia* and supergroup F *Wolbachia* strains based on MLST data are shown in (**b**,**c**), respectively. Strains that differ in a single mutation are connected with a solid line. Host information is provided in the white box with the number of host species indicated in parentheses.

MLST analysis indicated that some *Wolbachia* strains from ants and ant crickets shared identical or similar allelic profiles at MLST loci (Table 1, Figure 2). The MLST sequence type of *w*Msp6 (ST19) was identical to two *Wolbachia* strains (*w*LonA and *w*CamA1) detected from two ant host species of ant crickets*, P. longicornis* and *Camponotus* sp. (Table 1, Figure 2a,b). ST19 was widespread in ants as it has been found in eight strains from eight ant species (Figure 2b, species information was obtained from the PubMLST database). *w*Msp4 and *w*Msp7 (ST528) are virtually identical to two ant-associated STs, ST577 (from *Camponotus* sp.; *w*CamA3) and ST57 (from *Camponotus leonardi*; [32]), differing only by 1 bp across MLST loci (Figure 2a,b). ST471 was shared between host-specialist ant cricket, *M. americanus* (*w*Mame1) and its ant host *P. longicornis* (*w*LonF) (Figure 2a,c). 

The relationships among *Wolbachia* strains inferred by the *wsp* gene were similar, yet not completely congruent, to those inferred by MLST dataset. In some cases, strains that carry closely related MLST sequences possessed similar *wsp* sequences: *Wolbachia w*Msp6, *w*Msp4 and *w*Mame1 had *wsp* sequences identical to *Wolbachia w*LonA/*w*CamA1, *w*CamA3 and *w*LonF, respectively (Table 1, Table 2). On the other hand, some strains with similar *wsp* sequences could be divergent at MLST loci: *w*Msp2, *w*Msp3, *w*Mame1 and one *Wolbachia* strain, *w*Mul (*wsp*: MN044106; MLST: ST527), detected from parasitoid mites possessed closely related *wsp* sequences (percentage identity between all pairs of strain ranges from 99.23% to 100%; Table 2, also see Figure S2b for the *wsp* gene tree), while all these strains share either none or one identical MLST allele among each other with percentage identity between all pairs of strain ranging from 98.75% to 99.04%. 

## 4. Discussion

One major finding of this study is the extensive sharing of *Wolbachia* strains among ants and ant crickets, including three cases where ants and ant crickets share identical or nearly identical *Wolbachia* strains (*w*Mame1, *w*Msp4 and *w*Msp6). The *w*Mame1 (ST471) *Wolbachia* is only known from *M*. *americanus* and its exclusive host, *P*. *longicornis*. The *w*Msp6 (ST19) strain was shared between the host-generalist ant cricket *Myrmophilellus pilipes* and its ant host *P*. *longicornis* (and also *Camponotus* sp.). Surprisingly, we failed to detect *w*Msp4 (ST528) *Wolbachia* from the ant-crickets’ hosts (i.e., *P*. *longicornis* and *A*. *gracilipes*), yet found *w*Msp4-like *Wolbachia* in *Camponotus* sp.

Identical *Wolbachia* (*w*Mame1) shared between *M*. *americanus* and its ant host *P*. *longicornis* suggest the occurrence of horizontal transmission. Integrated host-specialists possess high degrees of host dependence, and acquire food exclusively via trophallaxis with ants [21,33,34], readily providing opportunities for interspecific transfer of *Wolbachia*. This pattern is consistent with the prediction in which social interactions may facilitate *Wolbachia* horizontal transmission between cohabiting species [17,18,19]. Nevertheless, the absence of shared *Wolbachia* between host-specialist *M. albicinctus* and its ant host *A. gracilipes* suggests cohabitation may not always result into successful *Wolbachia* horizontal transmission. 

*w*Msp6 (ST19) was shown to have a wide host range infecting hosts from three different insect orders (Hymenoptera, Lepidoptera and Coleoptera), implying inter-ordinal transfers of this *Wolbachia* may have occurred multiple times [6,32]. Our study not only expands the current knowledge of host range of *w*Msp6 (e.g., Orthoptera) but also indicates this strain in ant crickets likely results from inter-ordinal transfers from ant hosts, considering that *w*Msp6 was shared among ant crickets and ant hosts, and detected in only one ant cricket species with relatively low prevalence. Given that predation often serves as a route for *Wolbachia* horizontal transmission [12,14,15], host-generalist ant crickets may acquire novel *Wolbachia* strains through utilization of host ants as prey or stealing ant food [21,25,35,36].

It is noteworthy that the integrated host-specialists (e.g., *M*. *americanus* and *M*. *albicinctus*) tend to harbor higher *Wolbachia* prevalence and diversity among three types of ant cricket species. *w*Msp4 persists with a high frequency only in *M*. *americanus* (Figure 1b) but not in other *w*Msp4-infected ant cricket species, suggesting that *M*. *americanus* is particularly prone to *Wolbachia* infection. While the reasoning behind high diversity and prevalence of *Wolbachia* in integrated host-specialists remains unclear, one hypothesis is that the degree of host dependence may interact with *Wolbachia* persistence within host populations due to different selection forces operating on hosts, or factors related to a lifestyle beneficial for the establishment of *Wolbachia* (e.g., limited dispersal [37]). Support for this observation is available in the system involving fire ants and their social parasites in which an unexpectedly high *Wolbachia* diversity was found in the social parasites (up to eight strains), while the free-living hosts rarely harbor more than one *Wolbachia* strain [17,38]. However, one could also argue that these two sister species share common traits/genetic backgrounds encouraging the *Wolbachia* persistence in the populations.

We note that some *Wolbachia* strains in this study share similar *wsp* sequences but exhibit a comparatively low level of similarity at MLST loci (i.e., *w*Msp2, *w*Msp3, *w*Mame and *w*Mul, Figure S2, Table 2). This is perfectly in line with earlier studies where incongruence between the *wsp* gene and MLST is shown to be widespread in insects [3,39]. Furthermore, the finding of a high level of similarity at a fast evolving gene (i.e., *wsp*) but divergent at housekeeping genes (i.e., MLST) for these *Wolbachia* strains indicates that horizontal transfer of *wsp* sequences may have occurred through recombination [3,39]. It is speculated that the *wsp* participates in facilitating the early settlement and persistence of *Wolbachia* into a new host, and extensive horizontal gene transfer may have played one of key roles in adaptive evolution of this gene [40]. We argue that *Wolbachia* in ants and their associated myrmecophiles may represent a good study system to elucidate the role of the *wsp* during host exploitation.

In conclusion, our data suggest that horizontal transmission is key to explaining the distribution of *Wolbachia* across ant crickets. The shared *Wolbachia* among ant crickets and ant hosts indicates that kleptoparasitism represents an additional mechanism of inter-ordinal transfer of *Wolbachia*. Further *Wolbachia* surveys on species with similar kleptoparasitic nature may uncover the generality of this phenomenon as well as its underlying mechanisms. 

## Figures and Tables

**Table 1:** *wsp* accession number and multi-locus sequence type (MLST) allelic profiles of the *Wolbachia* strains recovered from the tested ant crickets and ants in this study.

Strain	Host Type	Host Species	*wsp* Accession No.	MLST id	ST ^a^	Supergroup	*gatB*	*coxA*	*hcpA*	*ftsZ*	*fbpA*
*w*Msp1	Ant cricket	*M. albicinctus*	MK995471	1926	525	A	294	291	331	251	459
*w*Msp2	Ant cricket	*M. quadrispina*	MK995472	1927	526	F	170	147	178	252	125
*w*Msp3	Ant cricket	*M. quadrispina*	MK995473	1928	526	F	170	147	178	252	125
*w*Msp4	Ant cricket	*M. americanus*, *M. albicinctus*, *M. hebardi*, *M. antilucanus*	MK995474	1929	528	A	49	44	297	42	49
*w*Msp5	Ant cricket	*M. americanus*, *M. quadrispina*	MK995475	1930	529	F	295	94	332	85	460
*w*Msp6	Ant cricket	*Myrmophilellus pilipes*	MK995476	1931	19	A	7	6	7	3	8
*w*Msp7	Ant cricket	*M. hebardi*	MK995477	1932	528	A	49	44	297	42	49
*w*Msp8	Ant cricket	*M. albicinctus*	MK995478	1933	531	A	49	44	333	253	49
*w*Mame1	Ant cricket	*M. americanus*	MK995479	2024	471 ^a^	F	168	147	262	132	226
*w*Mame2	Ant cricket	*M. americanus*	MK995480	NA	NA	NA	NA	NA	NA	NA	NA
*w*LonA	Ant	*Paratrechina longicornis*	MN927214	1827	19	A	7	6	7	3	8
*w*LonF	Ant	*Paratrechina longicornis*	MN927215	1828	471	F	168	147	262	132	226
*w*Agra	Ant	*Anoplolepis gracilipes*	MN927216	2017	52	A	22	2	51	32	36
*w*CamA1	Ant	*Camponotus* sp.	MN927217	2021	19 ^b^	A	7	6	7	3	8
*w*CamA2	Ant	*Camponotus* sp.	MN927218	2022	576 ^b^	A	323	2	47	45	481
*w*CamA3	Ant	*Camponotus* sp.	MN967009	2023	577 ^b^	A	49	44	297	42	482

^a^ ST: sequence type; ^b^ An inferred ST based on next-generation sequencing data and available allelic profile in the MLST database; NA: not applicable

**Table 2:** Comparisons of *wsp* sequences of *Wolbachia* from tested ant crickets and those in the GenBank and PubMLST databases.

Strain	GenBank Accession no./ PubMLST id	Percent Identity	Host (Strain)	Common Name
*w*Mame1	MN927215/ id: #1828	100%	*Paratrechina longicornis* (*w*LonF)	longhorn crazy ant
*w*Mame2	KC161941	97.22%	*Tachinid* sp.	tachinid fly
	KC161936	97.22%	*Pyralidid* sp.	pyralid moth
*w*Msp1	MG797608	99.63%	*Loxoblemmus equestris*	hard-headed cricket
*w*Msp2	MN044106	100%	*Macrodinychus multispinosus*	parasitoid mite
	MN927215/ id: #1828	99.62%	*Paratrechina longicornis* (*w*LonF)	longhorn crazy ant
*w*Msp3	MN044106	99.62%	*Macrodinychus multispinosus*	parasitoid mite
	MN927215/ id: #1828	99.23%	*Paratrechina longicornis* (*w*LonF)	longhorn crazy ant
*w*Msp4	MN967009	100%	*Camponotus* sp. (*w*CamA3)	carpenter ant
	KU527484	100%	*Tetramorium lanuginosum*	wooly ant
	KC137165	100%	*Odontomachus* sp.	trap jaw ant
	GU236978	100%	*Aulacophora nigripennis*	leaf beetle
	MG551859	100%	*Octodonta nipae*	nipa palm hispid beetle
	id: #120	100%	*Camponotus leonardi*	carpenter ant
*w*Msp5	KM078883	94.64%	*Chorthippus parallelus*	meadow grasshopper
	JN701984	94.41%	*Chorthippus parallelus*	meadow grasshopper
*w*Msp6	MN927214/ id: #1827	100%	*Paratrechina longicornis* (*w*LonA)	longhorn crazy ant
	MN927217	100%	*Camponotus* sp. (*w*CamA1)	carpenter ant
	KU527480	100%	*Tapinoma sessile*	odorous house ant
	KU527478	100%	*Tapinoma melanocephalum*	ghost ant
	HQ602874	100%	*Ceutorhynchus neglectus*	weevil
	AB024571	100%	*Ephestia cautella*	almond moth
	id: #111	100%	*Technomyrmex albipes*	white-footed ant
	id: #115	100%	*Leptomyrmex* sp.	spider ant
	id: #141	100%	*Pheidole* sp.	big-headed ant
	id: #146	100%	*Leptogenys* sp.	razorjaw ant
	id: #116	100%	*Myrmecorhynchus* sp.	ant
	id: #124	100%	*Pheidole plagiara*	big-headed ant
	id: #125	100%	*Pheidole sauberi*	big-headed ant
	id: #135	100%	*Ochetellus glaber*	black household ant
	id: #13	100%	*Ephestia kuehniella*	mediterranean flour moth
	id: #123	100%	*Ornipholidotos peucetia*	glasswings
	id: #451	100%	*Aricia artaxerxes*	northern brown argus
*w*Msp7	MN967009	99.81%	*Camponotus* sp. (*w*CamA3)	carpenter ant
	KU527484	99.81%	*Tetramorium lanuginosum*	wooly ant
	KC137165	99.81%	*Odontomachus* sp.	trap jaw ant
	GU236978	99.81%	*Aulacophora nigripennis*	leaf beetles
	MG551859	99.81%	*Octodonta nipae*	nipa palm hispid beetle
	id: #120	99.81%	*Camponotus leonardi*	carpenter ants
*w*Msp8	EF219194	95.47%	*Ixodes ricinus*	castor bean tick

## Supplementary Materials

The following are available online at https://www.mdpi.com/2076-2607/8/6/805/s1; Figure S1: Alignment of Illumina paired-end sequence reads to the reference MLST sequences, Figure S2: Genealogical relationships of *Wolbachia* strains based on the *wsp* gene, Table S1: Behavioral differences between non-integrated and integrated ant crickets based on Ooi (2019), Table S2: Recorded host ants of the tested ant cricket species, Table S3: Profile information of the ant cricket samples used in this study, Table S4: Primer sequences for the *wsp* gene and PCR conditions used in this study.
