# Supplementary material for: Evidence for Common Horizontal Transmission of Wolbachia among Ants and Ant Crickets: Kleptoparasitism Added to the List
Source: Microorganisms. 2020 May 27;8(6):805. doi: 10.3390/microorganisms8060805 (PMC7355411; doi:10.3390/microorganisms8060805)
Supplement: Supplementary file 1 [file microorganisms-08-00805-s001.pdf]

Supporting information

Supporting Methods and Results

Screening of the filarial nematodes in ant cricket

To rule out the possibility that the detected *Wolbachia* were derived from parasitic filarial nematodes frequently found inside insects [1], we screened for the presence of filarial nematodes using two polymerase chain reaction (PCR) assays involving two nematode universal primer pairs (5.8s-1/KK-28S-22 [2]; SSU18/SSU26R [3]) that amplify the ITS2 region and 18S RNA gene of nematodes, respectively. A PCR mixture was set up in a reaction volume of 25 µl using Takara EmeraldAmp Max PCR Master Mix (Takara, Japan). PCRs were carried out following the procedures described in main text with slight modifications (56 °C as annealing temperature for ITS2 region; 50 °C for 18S RNA gene). We detected no signs of filarial nematodes in any ant cricket samples in this study.

Wolbachia strain verification in *Myrmecophilus americanus* using Illumina sequence data

The *Wolbachia* MLST gene sequences of *M. americanus* were characterized using genome sequences generated by the high-throughput sequencing method. DNA libraries were prepared from genomic DNA of three *M. americanus* collected from Taiwan using the Truseq Nano DNA HT Sample Prep Kit (Illumina, USA) for 350 bp inserts, and each DNA library was sequenced on the Illumina Hiseq 4000 platform by Genomics BioSci and Tech Corp (Taipei, Taiwan), generating 150 bp paired-end reads. Trimmomatic 0.36 [4] was employed to remove adaptor sequences and trim bases with quality lower than 20 (QV20). We detected four types of *Wolbachia* infection in *M. americanus* by sequencing *wsp* gene from a number of clones. One type of *wsp* sequence, namely *w*Mame2, differed from all known *Wolbachia* strains. Three of the four types of *wsp* sequence in *M. americanus* were identical to *Wolbachia* strains *w*Msp4 and *w*Msp5 from ant crickets, and *w*LonF from longhorn crazy ant, *Paratrechina longicornis* (Fig. 1b, Table 2), respectively. To confirm *Wolbachia* strain identity detected in *M. americanus*, we mapped the sequencing reads of *M. americanus* onto the MLST reference sequences from *w*Msp4, *w*Msp5 and *w*LonF using bowtie2 v2.3.3 [5] in the local alignment

mode. The bowtie outputs (BAM files) were deposited in Zendo (doi: 10.5281/zenodo.3653448). *Wolbachia* type *w*Mame2 was excluded from the analysis because reference sequences of *w*Mame2-like *Wolbachia* were not available. Integrative Genomics Viewer (IGV version 2.5.3) was used to visualize the mapping [6]. Multiple reads matched with the entire reference sequences perfectly, with one exception: the reference sequence *fbpA* of *wLon*F was only partially aligned (Figure S1E). Therefore, a primer pair, [FbpwLonF-F (5′- GCTCCAATTCTTTGCATTCAA-3′) and FbpwLonF-R (5′-CCAATTCGTTTGGATAACGAT-3′)], was designed to amplify the *fbpA* sequence unique to *w*LonF in *M. americanus* samples. The PCR conditions included an initial denaturation step at 95°C (3 min) followed by 35 cycles of 94°C (30 s), 55°C (30 s), 72°C (1 min) and a final extension phase at 72°C (7 min). The PCR amplicons were purified and sequenced. A total of five *M. americanus* infected with *w*Mame1 were sequenced in both directions by using the specific primers. The results indicated that the sequences obtained from *M. americanus* were identical to the *fbpA* sequence of *w*LonF. Summing up, we conclude that the *Wolbachia* in *M. americanus* were identical to *w*Msp4, *w*Msp5 and *w*LonF at both *wsp* and MLST loci, assuming no sequence recombination among strains.

Determination of *Wolbachia* MLST alleles in *Camponotus* sp. triple infection samples

Results of *wsp* sequencing indicated that the ant samples from *Camponotus* sp. colony were infected with three types of *Wolbachia*, *w*CamA1, *w*CamA2 and *w*CamA3. The *wsp* sequences of *w*CamA1, and *w*CamA3 were identical to *w*Msp6 and *w*Msp4, while *w*CamA2 was identical to *Wolbachia* strains previously reported from ants *Rhytidoponera metallica* (Strain: Rmet_A; PubMLST id: #122) and *Pheidole* sp. (Phe_A_19-04; PubMLST id: #127). We utilized both nanopore-based sequencing and Sanger sequencing to characterize *Wolbachia* MLST allele profiles for these three *Wolbachia* strains. The PCR amplicons of *Wolbachia* MLST genes were purified by using the Gel/PCR Extraction Kit (Nippon Gene, Japan) and 1 mg of purified DNA (200 ng for each gene) was used to construct nanopore sequencing library using the Oxford Nanopore Technologies SQK-LSK109 kit following the manufacturer's instructions. Sequencing was performed on an Oxford Nanopore Technologies (Oxford, UK) MinION (MIN-101B) with FLO-MINSP6 flow cell. The sequencing run was terminated after 2 h and generated 449,808 reads. Reads with an average Q-score lower than 10 or of a length

smaller than 360 bp were filtered out through NanoFilt v 2.6.0 [7], resulting in 24,674 remaining sequences. We mapped all the filtered sequencing reads onto the MLST reference sequences from *w*Msp6 using bowtie2 v2.3.3 [5] in the local alignment mode. The bowtie outputs (BAM files) were deposited in Zendo (doi: 10.5281/zenodo.3653448). Integrative Genomics Viewer (IGV version 2.5.3) was used to visualize the mapping results and recover MLST haplotypes with SNPs that are identified in the mapping results [6]. All the SNPs detected by mapping were validated by bi-directional Sanger sequencing of the five MLST loci. The mapping result indicated that the five MLST loci were represented by three alleles each, and allele types were identical or nearly identical to MLST alleles found in one of the following *w*Msp4, *w*Msp6 or Phe_A_19-04 (1 bp difference from *w*Msp4 at *fbpA*; 1 bp difference from Phe_A_19-04 at *gatB* and *fbpA*). The MLST allele profile of *w*CamA1, *w*CamA2 and *w*CamA3 was constructed based on the strain information of *w*Msp4, *w*Msp6 or Phe_A_19-04, assuming no sequence recombination among strains.

Reconstruction of *wsp* gene tree

We estimated the *wsp* gene tree using a maximum-likelihood (ML) method with RAxML Blackbox web-servers [8]. All available *Wolbachia* sequences from orthopteran insects and ants were downloaded from GenBank and *Wolbachia* PubMLST database and included in the analysis after excluding redundant sequences. Alignment of *wsp* dataset was constructed on the GUIDANCE2 Server [9] based on codons using the MAFFT algorithm [10], and ambiguous alignments with the confidence score below 0.7 were excluded (426 bp remained). The nucleotide substitution models and best partitioning schemes were estimated with PartitionFinder version 2.1.1 [11] using the Akaike information criterion and a heuristic search algorithm. The best partitioning scheme selected by PartitionFinder for *wsp* gene were data partitioned by codon positions under the GTR+I+G model of rate substitution.

References

1. Fox, L.M. Blood and tissue nematodes: Filarial Worms. In *Principles and Practice of Pediatric Infectious Diseases*, 5th ed. Amsterdam, the Netherlands: Elsevier; **2017**. pp. 1388-1394.e1.
2. Barrière, A.; Félix, M.-A. Isolation of *C. elegans* and Related Nematodes. In *Wormbook* **2006**, *2,* 1–19.
3. Floyd, R.; Abebe, E.; Papert, A.; Blaxter, M. Molecular barcodes for soil nematode identification. *Mol. Ecol.* **2002**, *11*, 839–850, doi:10.1046/j.1365-294X.2002.01485.x.
4. Bolger, A.M.; Lohse, M.; Usadel, B. Trimmomatic: a flexible trimmer for Illumina sequence data. *Bioinformatics* **2014**, *30*, 2114–2120, doi:10.1093/bioinformatics/btu170.
5. Langmead, B.; Salzberg, S.L. Fast gapped-read alignment with Bowtie 2. *Nat. Methods* **2012**, *9*, 357, doi:10.1038/nmeth.1923.
6. Robinson, J.T.; Thorvaldsdóttir, H.; Wenger, A.M.; Zehir, A.; Mesirov, J.P. Variant review with the integrative genomics viewer. *Cancer Res*. **2017**, *77*, e31-e34, doi: 10.1158/0008-5472.CAN-17-0337.
7. De Coster, W.; D'Hert, S.; Schultz, D.T.; Cruts, M.; Van Broeckhoven, C. NanoPack: visualizing and processing long-read sequencing data. *Bioinformatics* **2018**, *34*, 2666–2669, doi: 10.1093/bioinformatics/bty149.
8. Kozlov, A.; Darriba, D.; Flouri, T.; Morel, B.; Stamatakis, A. RAxML-NG: a fast; scalable and user-friendly tool for maximum likelihood phylogenetic inference. **2019**, https://doi.org/10.1093/bioinformatics/btz305 available at: https://raxml-ng.vital-it.ch/#/
9. Penn, O.; Privman, E.; Ashkenazy, H.; Landan, G.; Graur, D.; Pupko, T. GUIDANCE: a web server for assessing alignment confidence scores. *Nucleic Acids Res.* **2010**, *38,* W23–W28, doi:10.1093/nar/gkq443.
10. Katoh, K.; Standley, D.M. MAFFT multiple sequence alignment software version 7: Improvements in performance and usability. *Mol. Biol. Evol.* **2013**, *30,* 772–780, doi:10.1093/molbev/mst010.
11. Lanfear, R.; Calcott, B.; Ho, S.Y.W.; Guindon, S. PartitionFinder: combined selection of partitioning schemes and substitution models for phylogenetic snalyses. *Mol. Biol. Evol.* **2012**, *29,* 1695–1701, doi:10.1093/molbev/mss020.
12. Ooi M. Integration, morphological differences and behavioural adaptations of ant crickets from the family Myrmecophilidae in association with host ants, *Paratrechina longicornis* and *Anoplolepis gracilipes* (Hymenoptera: Formicidae) (Unpublished master's thesis). **2019**, School of Biological Sciences, Universiti Sains Malaysia, Malaysia.

13. Komatsu, T.; Maruyama, M.; Itino, T. Behavioral differences between two ant cricket species in Nansei islands: Host-specialist versus host-generalist. *Insectes Soc.* **2009**, *56*, 389–396, doi:10.1007/s00040-009-0036-y.
14. Maruyama, M. Family Myrmecophilidae Saussure, 1870. *In: Orthopterological Society of Japan.* (Ed.) Orthoptera of the Japanese Archipelago in Color. Hokkaido University Press, Hokkaido, **2006**, 490–492 pp.
15. Wetterer, J.K.; Huge, S. Worldwide spread of the ant cricket *Myrmecophilus americanus*, a symbiont of the longhorn crazy ant, *Paratrechina longicornis*. *Sociobiology* **2008**, *52,* 157–165.
16. Hsu, P.W.; Hugel, S.; Wetterer, J.K.; Tseng, S.P.; Ooi, C.S.M.; Lee, Y.; Yang, C.C.S. Ant crickets (Orthoptera: Myrmecophilidae) associated with the invasive yellow crazy ant *Anoplolepis gracilipes* (Hymenoptera: Formicidae): evidence for cryptic species and potential co-introduction with hosts. *Myrmecol. News* **2020**, *30*: 103–129, doi: 10.25849/myrmecol.news_030:103.
17. Wasmann, E. Kritisches Verzeichniss der Myrmekophilen und Termitophilen Arthropoden: Mit Angabe der Lebensweise und mit Beschreibung neuer Arten. Verlag Von Felix L. Dames, Berlin. **1894**, 231 pp.
18. Kistner, D.H.; Chong, K.F.; Lee, C.Y. A new Malaysian *Myrmecophilous* cricket (Orthoptera: Myrmecophilidae). *Sociobiology* **2007**, *50*, 173–182.
19. Mann, W.M. Ant guests from Fiji and the British Solomon Islands. *Ann. Entomol. Soc. Am.* **1920**, *13*, 60–69, doi:10.1093/aesa/13.1.60.
20. Komatsu, T.; Maruyama, M.; Ueda, S.; Itino, T. MtDNA phylogeny of Japanese ant crickets (Orthoptera: Myrmecophilidae): Diversification in host specificity and habitat use. *Sociobiology* **2008**, *52*, 553–565.
21. Desutter-Grandcolas, L. First record of ant-loving crickets (Orthoptera: Myrmecophilidae: Myrmecophilinae) in New Caledonia. *Aust. J. Entomol.* **1997**, *36*, 159–163, doi:10.1111/j.1440-6055.1997.tb01449.x.
22. Komatsu, T.; Maruyama, M. Additional records of the distribution and host ant species for the ant cricket *Myrmophilellus pilipes*. *Insectes Soc.* **2016**, *63*, 623–627, doi:10.1007/s00040-016-0496-9.
23. Zhou, W.; Rousset, F.; O'Neill, S. Phylogeny and PCR–based classification of *Wolbachia* strains using *wsp* gene sequences. *Proc. R. Soc. London. Ser. B Biol. Sci.* **1998**, *265*, 509–515, doi:10.1098/rspb.1998.0324.

Table S1 Behavioral differences between non-integrated and integrated ant crickets based on Ooi (2019) [12]

	Non-integrated ant crickets	Integrated ant crickets
Preferred location in ant nest	Peripheral	Within nest
Feeding	Feed on food without the aid of host ants	Feed via mouth-to-mouth trophallaxis by ant worker
Trophallaxis	Unable to initiate the trophallaxis with host ants, but sometimes interrupt trophallaxis by two ant workers to take the food being transferred	Able to initiate trophallaxis with host ants
Consume ant brood	Often	Rare
Interaction with ant host	Avoid contact with ant and escape rapidly by running and jumping	Come into contact with an ant worker and groom it, licking the body of the ant
Level of host dependence	Medium to low, able to survive more than 2 months without host ants	High, unable to survive more than 1 month without host ants

Table S2 Recorded host ants of the tested ant cricket species

Recorded hosts	List of host ant species	References
Myrmecophilus albicinctus	*Anoplolepis gracilipes* (major), *Pheidole* sp. (one record)	[13, 14]
Myrmecophilus americanus	*Paratrechina longicornis* (major), *Camponotus* sp. (one record)	[15]
Myrmecophilus antilucanus	*Anoplolepis gracilipes*	[16]
Myrmecophilus dubius	*Anoplolepis gracilipes* (major)	[16, 17]
Myrmecophilus hebardi	*Paratrechina longicornis* (major), *Camponotus* sp. (one record)	[16, 18, 19]
Myrmecophilus quadrispina	*Anoplolepis gracilipes, Paratrechina longicornis, Solenopsis invicta, Solenopsis geminata, Pheidole megacephala, Carebara diversus, Polyrhachis dives, Nylanderia amia, Camponotus kaguya, Pheidole noda, Pheidole parva, Pheidole* sp., *Diacamma* sp., *Brachyponera chinensis*	[16, 13, 20, 21]
Myrmophilellus pilipes	*Anoplolepis gracilipes, Paratrechina longicornis*, *Camponotus* sp., *Diacamma* spp., *Pheidole megacephala, Pheidole* sp., *Solenopsis geminata, Dolichoderus thoracicus, Carebara diversus, Proatta butteli, Philidris cordata*	[16, 22]

Table S3 Profile information of the ant cricket samples used in this study

Sample	Species	*Wolbachia* strain	Host ant species	Host *Wolbachia* type	Locality	
AnoTH04C04	*Myrmecophilus albicinctus*	*w*Msp4, *w*Msp8	*Anoplolepis gracilipes*	*w*Agra	Thailand	Nong Sarai, Pak Chong District
AnoBOT01C03	*Myrmecophilus albicinctus*	*w*Msp1	*Anoplolepis gracilipes*	*w*Agra	Malaysia	Botanical Garden, Jalan Kebun Bunga, Penang
AnoKIC03	*Myrmecophilus albicinctus*	*w*Msp1	*Anoplolepis gracilipes*	*w*Agra	Malaysia	Penang National Park, Pulau Pinang
Anomy35C01	*Myrmecophilus albicinctus*	*w*Msp1	*Anoplolepis gracilipes*	Uninfected	Malaysia	Aman Hostel, USM
Anomy36C02	*Myrmecophilus albicinctus*	*w*Msp1	*Anoplolepis gracilipes*	*w*Agra	Malaysia	Subaidah,USM
Anomy36C03	*Myrmecophilus albicinctus*	*w*Msp1	*Anoplolepis gracilipes*	*w*Agra	Malaysia	Subaidah,USM
Ano84.C01	*Myrmecophilus albicinctus*	*w*Msp1	*Anoplolepis gracilipes*	*w*Agra	Taiwan	Daxi Dist., Taoyuan City
Ano84.C02	*Myrmecophilus albicinctus*	*w*Msp1	*Anoplolepis gracilipes*	*w*Agra	Taiwan	Daxi Dist., Taoyuan City
Ano84.C03	*Myrmecophilus albicinctus*	*w*Msp1	*Anoplolepis gracilipes*	*w*Agra	Taiwan	Daxi Dist., Taoyuan City
Ano84.C04	*Myrmecophilus albicinctus*	*w*Msp1	*Anoplolepis gracilipes*	*w*Agra	Taiwan	Daxi Dist., Taoyuan City
Ano125C02	*Myrmecophilus albicinctus*	*w*Msp1, *w*Msp8	*Anoplolepis gracilipes*	NA	Taiwan	Taitung Dawu Township
Ano125C04	*Myrmecophilus albicinctus*	*w*Msp1, *w*Msp8	*Anoplolepis gracilipes*	NA	Taiwan	Taitung Dawu Township
Ano125C05	*Myrmecophilus albicinctus*	*w*Msp1, *w*Msp8	*Anoplolepis gracilipes*	NA	Taiwan	Taitung Dawu Township
Ano125C06	*Myrmecophilus albicinctus*	*w*Msp1, *w*Msp8	*Anoplolepis gracilipes*	NA	Taiwan	Taitung Dawu Township
Ano125C08	*Myrmecophilus albicinctus*	*w*Msp1, *w*Msp8	*Anoplolepis gracilipes*	NA	Taiwan	Taitung Dawu Township
Ano125C09	*Myrmecophilus albicinctus*	*w*Msp1, *w*Msp8	*Anoplolepis gracilipes*	NA	Taiwan	Taitung Dawu Township
AgrJP18.4C1	*Myrmecophilus albicinctus*	*w*Msp4, *w*Msp8	*Anoplolepis gracilipes*	NA	Japan	Ryudai, Nakagami District, Okinawa
AnoJP46C02	*Myrmecophilus albicinctus*	*w*Msp4, *w*Msp8	*Anoplolepis gracilipes*	*w*Agra	Japan	Onna-son, Kunigami-gun, Okinawa
AnoJP47C04	*Myrmecophilus albicinctus*	*w*Msp4, *w*Msp8	*Anoplolepis gracilipes*	*w*Agra	Japan	Onna-son, Kunigami-gun, Okinawa

AnoJP48C01	*Myrmecophilus albicinctus*	*w*Msp4, *w*Msp8	*Anoplolepis gracilipes*	*w*Agra	Japan	Onna-son, Kunigami-gun, Okinawa
AnoJP49C02	*Myrmecophilus albicinctus*	*w*Msp4, *w*Msp8	*Anoplolepis gracilipes*	*w*Agra	Japan	Onna-son, Kunigami-gun, Okinawa
AnoTH04C02	*Myrmecophilus albicinctus*	*w*Msp4, *w*Msp8	*Anoplolepis gracilipes*	Uninfected	Thailand	Nong Sarai, Pak Chong District
AnoNT01c02	*Myrmecophilus albicinctus*	*w*Msp8	*Anoplolepis gracilipes*	*w*Agra	Taiwan	Chushan Township, Nantou County
AnoNT01c04	*Myrmecophilus albicinctus*	*w*Msp8	*Anoplolepis gracilipes*	*w*Agra	Taiwan	Chushan Township, Nantou County
AnoNT01c06	*Myrmecophilus albicinctus*	*w*Msp8	*Anoplolepis gracilipes*	*w*Agra	Taiwan	Chushan Township, Nantou County
AnoNT01c09	*Myrmecophilus albicinctus*	*w*Msp8	*Anoplolepis gracilipes*	*w*Agra	Taiwan	Chushan Township, Nantou County
AnoNT01c11	*Myrmecophilus albicinctus*	*w*Msp8	*Anoplolepis gracilipes*	*w*Agra	Taiwan	Chushan Township, Nantou County
AnoNT01c12	*Myrmecophilus albicinctus*	*w*Msp8	*Anoplolepis gracilipes*	*w*Agra	Taiwan	Chushan Township, Nantou County
AnoBOT01C01	*Myrmecophilus albicinctus*	Uninfected	*Anoplolepis gracilipes*	*w*Agra	Malaysia	Botanical Garden, Jalan Kebun Bunga, Penang
AnoKIC04	*Myrmecophilus albicinctus*	Uninfected	*Anoplolepis gracilipes*	*w*Agra	Malaysia	Penang National Park, Pulau Pinang
AnoKIC06	*Myrmecophilus albicinctus*	Uninfected	*Anoplolepis gracilipes*	*w*Agra	Malaysia	Penang National Park, Pulau Pinang
Anomy35C09	*Myrmecophilus albicinctus*	Uninfected	*Anoplolepis gracilipes*	Uninfected	Malaysia	Aman Hostel, USM
Anomy35C10	*Myrmecophilus albicinctus*	Uninfected	*Anoplolepis gracilipes*	Uninfected	Malaysia	Aman Hostel, USM
Anomy35C11	*Myrmecophilus albicinctus*	Uninfected	*Anoplolepis gracilipes*	Uninfected	Malaysia	Aman Hostel, USM
Anomy35C12	*Myrmecophilus albicinctus*	Uninfected	*Anoplolepis gracilipes*	Uninfected	Malaysia	Aman Hostel, USM
mal20	*Myrmecophilus albicinctus*	Uninfected	*Anoplolepis gracilipes*	NA	Malaysia	Pulau Pinang

mal80	*Myrmecophilus albicinctus*	Uninfected	*Anoplolepis gracilipes*	NA	Malaysia	Pulau Pinang
Ano84.C05	*Myrmecophilus albicinctus*	Uninfected	*Anoplolepis gracilipes*	*w*Agra	Taiwan	Daxi Dist., Taoyuan City
07.323-1	*Myrmecophilus americanus*	*w*Msp4, *w*Mame2	*Paratrechina longicornis*	Uninfected	Antigua	Darkwood Beach
14.489-1	*Myrmecophilus americanus*	*w*Msp4, *w*Msp5, *w*Mame1, *w*Mame2	*Paratrechina longicornis*	*w*LonA, *w*LonF	Singapore	City Hall
12.358-10	*Myrmecophilus americanus*	*w*Msp4, *w*Msp5, *w*Mame1, *w*Mame2	*Paratrechina longicornis*	*w*LonA	USA	Virginia Key, FL
12.358-11	*Myrmecophilus americanus*	*w*Msp4, *w*Msp5, *w*Mame1, *w*Mame2	*Paratrechina longicornis*	*w*LonA	USA	Virginia Key, FL
08.818-1	*Myrmecophilus americanus*	*w*Msp4, *w*Msp5, *w*Mame1, *w*Mame2	*Paratrechina longicornis*	*w*LonF	USA	Big Pine Key, FL
08.709-3	*Myrmecophilus americanus*	*w*Msp4, *w*Msp5, *w*Mame1, *w*Mame2	*Paratrechina longicornis*	Uninfected	Bonaire	Kralendijk
08.745-1	*Myrmecophilus americanus*	*w*Msp4, *w*Msp5, *w*Mame1, *w*Mame2	*Paratrechina longicornis*	Uninfected	Bonaire	Belnem
11.543-1	*Myrmecophilus americanus*	*w*Msp4, *w*Msp5, *w*Mame1, *w*Mame2	*Paratrechina longicornis*	*w*LonF	Curaçao	Juan Domingo
11.368-1	*Myrmecophilus americanus*	*w*Msp4, *w*Msp5, *w*Mame1, *w*Mame2	*Paratrechina longicornis*	*w*LonF	Curaçao	Playa Forti
11.507-2	*Myrmecophilus americanus*	*w*Msp4, *w*Msp5, *w*Mame1, *w*Mame2	*Paratrechina longicornis*	Uninfected	Curaçao	Koredor
11.24-1	*Myrmecophilus americanus*	*w*Msp4, *w*Msp5, *w*Mame1, *w*Mame2	*Paratrechina longicornis*	Uninfected	Guadeloupe	Carénage
10.454-1	*Myrmecophilus americanus*	*w*Msp4, *w*Msp5, *w*Mame1, *w*Mame2	*Paratrechina longicornis*	*w*LonA	Jamaica	Negril
plmy89Mame01	*Myrmecophilus americanus*	*w*Msp4, *w*Msp5, *w*Mame1, *w*Mame2	*Paratrechina longicornis*	*w*LonF	Malaysia	Pulau Pinang
plmy89Mame02	*Myrmecophilus americanus*	*w*Msp4, *w*Msp5, *w*Mame1, *w*Mame2	*Paratrechina longicornis*	*w*LonF	Malaysia	Pulau Pinang

plmy89Mame03	*Myrmecophilus americanus*	*w*Msp4, *w*Msp5, *w*Mame1, *w*Mame2	*Paratrechina longicornis*	*w*LonF	Malaysia	Pulau Pinang
11.268-1	*Myrmecophilus americanus*	*w*Msp4, *w*Msp5, *w*Mame1, *w*Mame2	*Paratrechina longicornis*	*w*LonA	Martinique	Le Marin
hug88	*Myrmecophilus americanus*	*w*Msp4, *w*Msp5, *w*Mame1, *w*Mame2	*Paratrechina longicornis*	*w*LonF	Martinique	Spoutourne
07.642-1	*Myrmecophilus americanus*	*w*Msp4, *w*Msp5, *w*Mame1, *w*Mame2	*Paratrechina longicornis*	Uninfected	Montserrat	Brades
07.561-1	*Myrmecophilus americanus*	*w*Msp4, *w*Msp5, *w*Mame1, *w*Mame2	*Paratrechina longicornis*	*w*LonF	Montserrat	Brades
plTH22Mame1	*Myrmecophilus americanus*	*w*Msp4, *w*Msp5, *w*Mame1, *w*Mame2	*Paratrechina longicornis*	*w*LonF	Thailand	Wiset Chai Chan Dist, Ang Thong Province
plTH29Mame1	*Myrmecophilus americanus*	*w*Msp4, *w*Msp5, *w*Mame1, *w*Mame2	*Paratrechina longicornis*	*w*LonA	Thailand	Ban Pom, Phra Nakhon Si Ayutthaya District
12.356-1	*Myrmecophilus americanus*	*w*Msp4, *w*Msp5, *w*Mame1, *w*Mame2	*Paratrechina longicornis*	*w*LonF	USA	Rickenbacker Causeway, Miami, FL
12.391-1	*Myrmecophilus americanus*	*w*Msp4, *w*Msp5, *w*Mame1, *w*Mame2	*Paratrechina longicornis*	*w*LonA, *w*LonF	USA	Lake Worth, FL
14.376-1	*Myrmecophilus americanus*	*w*Msp4, *w*Msp5, *w*Mame2	*Paratrechina longicornis*	*w*LonA, *w*LonF	Singapore	City Hall
08.813-1	*Myrmecophilus americanus*	*w*Msp4, *w*Msp5, *w*Mame2	*Paratrechina longicornis*	Uninfected	USA	Key West, FL
07.359-1	*Myrmecophilus americanus*	*w*Msp4, *w*Msp5, *w*Mame2	*Paratrechina longicornis*	*w*LonF	Antigua	Boons Bay
07.507-2	*Myrmecophilus americanus*	*w*Msp4, *w*Msp5, *w*Mame2	*Paratrechina longicornis*	*w*LonF	Antigua	Long Bay
07.815-1	*Myrmecophilus americanus*	*w*Msp4, *w*Msp5, *w*Mame2	*Paratrechina longicornis*	Uninfected	Aruba	Cas di Paloma
10.62-1	*Myrmecophilus americanus*	*w*Msp4, *w*Msp5, *w*Mame2	*Paratrechina longicornis*	*w*LonF	Bahamas	New Providence, Coral Harbour

08.694-1	*Myrmecophilus americanus*	*w*Msp4, *w*Msp5, *w*Mame2	*Paratrechina longicornis*	*w*LonF	Bonaire	Bezu
08.709-4	*Myrmecophilus americanus*	*w*Msp4, *w*Msp5, *w*Mame2	*Paratrechina longicornis*	Uninfected	Bonaire	Kralendijk
10.303-1	*Myrmecophilus americanus*	*w*Msp4, *w*Msp5, *w*Mame2	*Paratrechina longicornis*	Uninfected	Jamaica	Montego Bay
hug087	*Myrmecophilus americanus*	*w*Msp4, *w*Msp5, *w*Mame2	*Paratrechina longicornis*	Uninfected	Martinique	Tartane
hug101	*Myrmecophilus americanus*	*w*Msp4, *w*Msp5, *w*Mame2	*Paratrechina longicornis*	Uninfected	Martinique	Le Robert
07.382-1	*Myrmecophilus americanus*	*w*Msp4, *w*Msp5, *w*Mame2	*Paratrechina longicornis*	*w*LonF	St. Martin	Airport Road
MameTw01-1	*Myrmecophilus americanus*	*w*Msp4, *w*Msp5, *w*Mame2	*Paratrechina longicornis*	NA	Taiwan	Dacun Township, Changhua County
MameTw01-2	*Myrmecophilus americanus*	*w*Msp4, *w*Msp5, *w*Mame2	*Paratrechina longicornis*	NA	Taiwan	Dacun Township, Changhua County
MameTw01-3	*Myrmecophilus americanus*	*w*Msp4, *w*Msp5, *w*Mame2	*Paratrechina longicornis*	NA	Taiwan	Dacun Township, Changhua County
MameMyn01	*Myrmecophilus americanus*	*w*Msp4, *w*Msp5, *w*Mame2	*Paratrechina longicornis*	*w*LonA, *w*LonF	Taiwan	Taichung
MameMyn02	*Myrmecophilus americanus*	*w*Msp4, *w*Msp5, *w*Mame2	*Paratrechina longicornis*	*w*LonA, *w*LonF	Taiwan	Taichung
Ano85.C01	*Myrmecophilus antilucanus*	*w*Msp4	*Anoplolepis gracilipes*	*w*Agra	Taiwan	Da'an Dist., Taipei City
Ano97C01	*Myrmecophilus antilucanus*	*w*Msp4	*Anoplolepis gracilipes*	*w*Agra	Taiwan	Sanwan Township, Miaoli County
Ano97C02	*Myrmecophilus antilucanus*	*w*Msp4	*Anoplolepis gracilipes*	*w*Agra	Taiwan	Sanwan Township, Miaoli County
Ano105C01	*Myrmecophilus antilucanus*	*w*Msp4	*Anoplolepis gracilipes*	*w*Agra	Taiwan	Daan Dist. Taipei
Ano105C02	*Myrmecophilus antilucanus*	*w*Msp4	*Anoplolepis gracilipes*	*w*Agra	Taiwan	Daan Dist. Taipei

Ano36.2C01	*Myrmecophilus antilucanus*	Uninfected	*Anoplolepis gracilipes*	*w*Agra	Malaysia	Subaidah, Gelugor, Pulau Pinang
Ano36.2C04	*Myrmecophilus antilucanus*	Uninfected	*Anoplolepis gracilipes*	*w*Agra	Malaysia	Subaidah, Gelugor, Pulau Pinang
Ano36.2C05	*Myrmecophilus antilucanus*	Uninfected	*Anoplolepis gracilipes*	*w*Agra	Malaysia	Subaidah, Gelugor, Pulau Pinang
Ano36.2C06	*Myrmecophilus antilucanus*	Uninfected	*Anoplolepis gracilipes*	*w*Agra	Malaysia	Subaidah, Gelugor, Pulau Pinang
Ano36.2C10	*Myrmecophilus antilucanus*	Uninfected	*Anoplolepis gracilipes*	*w*Agra	Malaysia	Subaidah, Gelugor, Pulau Pinang
mun1-r10	*Myrmecophilus antilucanus*	Uninfected	*Anoplolepis gracilipes*	NA	Malaysia	Pulau Pinang
mun1-r11	*Myrmecophilus antilucanus*	Uninfected	*Anoplolepis gracilipes*	NA	Malaysia	Pulau Pinang
mun1-r14	*Myrmecophilus antilucanus*	Uninfected	*Anoplolepis gracilipes*	NA	Malaysia	Pulau Pinang
mun1-r3	*Myrmecophilus antilucanus*	Uninfected	*Anoplolepis gracilipes*	NA	Malaysia	Pulau Pinang
mun1-r6	*Myrmecophilus antilucanus*	Uninfected	*Anoplolepis gracilipes*	NA	Malaysia	Pulau Pinang
mun1-r7	*Myrmecophilus antilucanus*	Uninfected	*Anoplolepis gracilipes*	NA	Malaysia	Pulau Pinang
mun1-r8	*Myrmecophilus antilucanus*	Uninfected	*Anoplolepis gracilipes*	NA	Malaysia	Pulau Pinang
mun1-r9	*Myrmecophilus antilucanus*	Uninfected	*Anoplolepis gracilipes*	NA	Malaysia	Pulau Pinang
UnknowC01	*Myrmecophilus antilucanus*	Uninfected	*Anoplolepis gracilipes*	NA	Malaysia	Pulau Pinang
Ano95C01	*Myrmecophilus antilucanus*	Uninfected	*Anoplolepis gracilipes*	*w*Agra	Taiwan	Hsinpu town, Hsinchu County
Ano95C02	*Myrmecophilus antilucanus*	Uninfected	*Anoplolepis gracilipes*	*w*Agra	Taiwan	Hsinpu town, Hsinchu County
Ano95C03	*Myrmecophilus antilucanus*	Uninfected	*Anoplolepis gracilipes*	*w*Agra	Taiwan	Hsinpu town, Hsinchu County
Ano95C04	*Myrmecophilus antilucanus*	Uninfected	*Anoplolepis gracilipes*	*w*Agra	Taiwan	Hsinpu town, Hsinchu County
Ano95C05	*Myrmecophilus antilucanus*	Uninfected	*Anoplolepis gracilipes*	*w*Agra	Taiwan	Hsinpu town, Hsinchu County

Ano95C06	*Myrmecophilus antilucanus*	Uninfected	*Anoplolepis gracilipes*	*w*Agra	Taiwan	Hsinpu town, Hsinchu County
AnoTH04C03	*Myrmecophilus antilucanus*	Uninfected	*Anoplolepis gracilipes*	*w*Agra	Thailand	Nong Sarai, Pak Chong District
AnoBOT01C02	*Myrmecophilus dubius*	Uninfected	*Anoplolepis gracilipes*	*w*Agra	Malaysia	Botanical Garden, Jalan Kebun Bunga, Penang
AnoBOT01C04	*Myrmecophilus dubius*	Uninfected	*Anoplolepis gracilipes*	*w*Agra	Malaysia	Botanical Garden, Jalan Kebun Bunga, Penang
AnoBOT01C05	*Myrmecophilus dubius*	Uninfected	*Anoplolepis gracilipes*	*w*Agra	Malaysia	Botanical Garden, Jalan Kebun Bunga, Penang
AnoBTG01	*Myrmecophilus dubius*	Uninfected	*Anoplolepis gracilipes*	*w*Agra	Malaysia	Botanical Garden, Jalan Kebun Bunga, Penang
AnoBTG02	*Myrmecophilus dubius*	Uninfected	*Anoplolepis gracilipes*	*w*Agra	Malaysia	Botanical Garden, Jalan Kebun Bunga, Penang
AnoBTG03	*Myrmecophilus dubius*	Uninfected	*Anoplolepis gracilipes*	*w*Agra	Malaysia	Botanical Garden, Jalan Kebun Bunga, Penang
AnoBTG04	*Myrmecophilus dubius*	Uninfected	*Anoplolepis gracilipes*	*w*Agra	Malaysia	Botanical Garden, Jalan Kebun Bunga, Penang
AnoBTG05	*Myrmecophilus dubius*	Uninfected	*Anoplolepis gracilipes*	*w*Agra	Malaysia	Botanical Garden, Jalan Kebun Bunga, Penang
AnoBTG06	*Myrmecophilus dubius*	Uninfected	*Anoplolepis gracilipes*	*w*Agra	Malaysia	Botanical Garden, Jalan Kebun Bunga, Penang
AnoKIC01	*Myrmecophilus dubius*	Uninfected	*Anoplolepis gracilipes*	*w*Agra	Malaysia	Penang National Park, Pulau Pinang
AnoKIC02	*Myrmecophilus dubius*	Uninfected	*Anoplolepis gracilipes*	*w*Agra	Malaysia	Penang National Park, Pulau Pinang
AnoKIC05	*Myrmecophilus dubius*	Uninfected	*Anoplolepis gracilipes*	*w*Agra	Malaysia	Penang National Park, Pulau Pinang
mpv-r1	*Myrmecophilus dubius*	Uninfected	*Anoplolepis gracilipes*	NA	Malaysia	Pulau Pinang
mpv-r10	*Myrmecophilus dubius*	Uninfected	*Anoplolepis gracilipes*	NA	Malaysia	Pulau Pinang

mpv-r11	*Myrmecophilus dubius*	Uninfected	*Anoplolepis gracilipes*	NA	Malaysia	Pulau Pinang
mpv-r2	*Myrmecophilus dubius*	Uninfected	*Anoplolepis gracilipes*	NA	Malaysia	Pulau Pinang
mpv-r5	*Myrmecophilus dubius*	Uninfected	*Anoplolepis gracilipes*	NA	Malaysia	Pulau Pinang
mpv-r6	*Myrmecophilus dubius*	Uninfected	*Anoplolepis gracilipes*	NA	Malaysia	Pulau Pinang
mpv-r8	*Myrmecophilus dubius*	Uninfected	*Anoplolepis gracilipes*	NA	Malaysia	Pulau Pinang
mpv-r9	*Myrmecophilus dubius*	Uninfected	*Anoplolepis gracilipes*	NA	Malaysia	Pulau Pinang
NP01	*Myrmecophilus dubius*	Uninfected	*Anoplolepis gracilipes*	NA	Malaysia	Pulau Pinang
NP02	*Myrmecophilus dubius*	Uninfected	*Anoplolepis gracilipes*	NA	Malaysia	Pulau Pinang
UnknowC03	*Myrmecophilus dubius*	Uninfected	*Anoplolepis gracilipes*	NA	Malaysia	Pulau Pinang
mp-r12	*Myrmecophilus hebardi*	*w*Msp4	*Anoplolepis gracilipes*	NA	Malaysia	Pulau Pinang
mp-r3	*Myrmecophilus hebardi*	*w*Msp4	*Anoplolepis gracilipes*	NA	Malaysia	Pulau Pinang
mp-r5	*Myrmecophilus hebardi*	*w*Msp4	*Anoplolepis gracilipes*	NA	Malaysia	Pulau Pinang
Anomy35C02	*Myrmecophilus hebardi*	*w*Msp7	*Anoplolepis gracilipes*	Uninfected	Malaysia	Aman Hostel, USM
Anomy35C03	*Myrmecophilus hebardi*	*w*Msp7	*Anoplolepis gracilipes*	Uninfected	Malaysia	Aman Hostel, USM
Anomy35C04	*Myrmecophilus hebardi*	*w*Msp7	*Anoplolepis gracilipes*	Uninfected	Malaysia	Aman Hostel, USM
Anomy35C05	*Myrmecophilus hebardi*	*w*Msp7	*Anoplolepis gracilipes*	Uninfected	Malaysia	Aman Hostel, USM
Anomy35C06	*Myrmecophilus hebardi*	*w*Msp7	*Anoplolepis gracilipes*	Uninfected	Malaysia	Aman Hostel, USM
Anomy36C01	*Myrmecophilus hebardi*	*w*Msp7	*Anoplolepis gracilipes*	*w*Agra	Malaysia	Subaidah,USM
Cam01C05	*Myrmecophilus hebardi*	*w*Msp7	*Camponotus* sp.	*w*CamA1, *w*CamA2, *w*CamA3	Malaysia	Lebuh Relau, Pulau Pinang
mp-r8	*Myrmecophilus hebardi*	*w*Msp7	*Anoplolepis gracilipes*	NA	Malaysia	Pulau Pinang
Ano17.09C01	*Myrmecophilus hebardi*	Uninfected	*Anoplolepis gracilipes*	*w*Agra	Taiwan	Hengchun Township, Pingtung County
Ano17.09C02	*Myrmecophilus hebardi*	Uninfected	*Anoplolepis gracilipes*	*w*Agra	Taiwan	Hengchun Township, Pingtung County
Ano17.10C03	*Myrmecophilus hebardi*	Uninfected	*Anoplolepis gracilipes*	*w*Agra	Taiwan	Hengchun Township, Pingtung County

Ano17.12C09	*Myrmecophilus hebardi*	Uninfected	*Anoplolepis gracilipes*	*w*Agra	Taiwan	Hengchun Township, Pingtung County
Ano40.C01	*Myrmecophilus hebardi*	Uninfected	*Anoplolepis gracilipes*	*w*Agra	Taiwan	Maolin Dist., Kaohsiung City
Anodyu01C01	*Myrmecophilus hebardi*	Uninfected	*Anoplolepis gracilipes*	Uninfected	Taiwan	Dacun Township, Changhua County
Anodyu01C02	*Myrmecophilus hebardi*	Uninfected	*Anoplolepis gracilipes*	Uninfected	Taiwan	Dacun Township, Changhua County
Anodyu01C03	*Myrmecophilus hebardi*	Uninfected	*Anoplolepis gracilipes*	Uninfected	Taiwan	Dacun Township, Changhua County
Anodyu01C04	*Myrmecophilus hebardi*	Uninfected	*Anoplolepis gracilipes*	Uninfected	Taiwan	Dacun Township, Changhua County
AnoNT01c03	*Myrmecophilus hebardi*	Uninfected	*Anoplolepis gracilipes*	*w*Agra	Taiwan	Chushan Township, Nantou County
AnoNT01c05	*Myrmecophilus hebardi*	Uninfected	*Anoplolepis gracilipes*	*w*Agra	Taiwan	Chushan Township, Nantou County
AnoNT01c07	*Myrmecophilus hebardi*	Uninfected	*Anoplolepis gracilipes*	*w*Agra	Taiwan	Chushan Township, Nantou County
AnoTH04C01	*Myrmecophilus hebardi*	Uninfected	*Anoplolepis gracilipes*	Uninfected	Thailand	Nong Sarai, Pak Chong District
AnoTH04C05	*Myrmecophilus hebardi*	Uninfected	*Anoplolepis gracilipes*	Uninfected	Thailand	Nong Sarai, Pak Chong District
AnoTH04C06	*Myrmecophilus hebardi*	Uninfected	*Anoplolepis gracilipes*	Uninfected	Thailand	Nong Sarai, Pak Chong District
pheton01.C01	*Myrmecophilus quadrispina*	*w*Msp2	*Carebara* sp. (*Pheidologeton*)	Uninfected	Taiwan	Shalu Dist., Taichung City
pheton01.C02	*Myrmecophilus quadrispina*	*w*Msp2	*Carebara* sp. (*Pheidologeton*)	Uninfected	Taiwan	Shalu Dist., Taichung City
pheton01.C04	*Myrmecophilus quadrispina*	*w*Msp2	*Carebara* sp. (*Pheidologeton*)	Uninfected	Taiwan	Shalu Dist., Taichung City
Pl330.C01	*Myrmecophilus quadrispina*	*w*Msp2, *w*Msp3	*Paratrechina longicornis*	*w*LonF	Taiwan	Taiping Dist., Taichung City

Ano95C20	*Myrmecophilus quadrispina*	*w*Msp5	*Anoplolepis gracilipes*	*w*Agra	Taiwan	Hsinpu town, Hsinchu County
Agr18.1C1	*Myrmecophilus quadrispina*	Uninfected	*Anoplolepis gracilipes*	NA	Taiwan	New Taipei City
Agr18.1C2	*Myrmecophilus quadrispina*	Uninfected	*Anoplolepis gracilipes*	NA	Taiwan	New Taipei City
Agr18.1C3	*Myrmecophilus quadrispina*	Uninfected	*Anoplolepis gracilipes*	NA	Taiwan	New Taipei City
Agr18.1C4	*Myrmecophilus quadrispina*	Uninfected	*Anoplolepis gracilipes*	NA	Taiwan	New Taipei City
Agr18.1C5	*Myrmecophilus quadrispina*	Uninfected	*Anoplolepis gracilipes*	NA	Taiwan	New Taipei City
Agr18.1C6	*Myrmecophilus quadrispina*	Uninfected	*Anoplolepis gracilipes*	NA	Taiwan	New Taipei City
Agr18.2C1	*Myrmecophilus quadrispina*	Uninfected	*Anoplolepis gracilipes*	NA	Taiwan	New Taipei City
Ano17.06C06	*Myrmecophilus quadrispina*	Uninfected	*Anoplolepis gracilipes*	*w*Agra	Taiwan	Hengchun Township, Pingtung County
Ano68.C01	*Myrmecophilus quadrispina*	Uninfected	*Anoplolepis gracilipes*	*w*Agra	Taiwan	Wulai Dist., New Taipei City
Ano68.C02	*Myrmecophilus quadrispina*	Uninfected	*Anoplolepis gracilipes*	*w*Agra	Taiwan	Wulai Dist., New Taipei City
Ano68.C03	*Myrmecophilus quadrispina*	Uninfected	*Anoplolepis gracilipes*	*w*Agra	Taiwan	Wulai Dist., New Taipei City
Ano68.C04	*Myrmecophilus quadrispina*	Uninfected	*Anoplolepis gracilipes*	*w*Agra	Taiwan	Wulai Dist., New Taipei City
Ano71.C01	*Myrmecophilus quadrispina*	Uninfected	*Anoplolepis gracilipes*	NA	Taiwan	Xiulin Township, Hualien County
Ano71.C02	*Myrmecophilus quadrispina*	Uninfected	*Anoplolepis gracilipes*	NA	Taiwan	Xiulin Township, Hualien County
Ano95C08	*Myrmecophilus quadrispina*	Uninfected	*Anoplolepis gracilipes*	*w*Agra	Taiwan	Hsinpu town, Hsinchu County
Ano97C03	*Myrmecophilus quadrispina*	Uninfected	*Anoplolepis gracilipes*	*w*Agra	Taiwan	Sanwan Township, Miaoli County
Ano97C04	*Myrmecophilus quadrispina*	Uninfected	*Anoplolepis gracilipes*	*w*Agra	Taiwan	Sanwan Township, Miaoli County

Phe17.01C03	*Myrmecophilus quadrispina*	Uninfected	*Pheidole* sp.	NA	Taiwan	Renai Township, Nantou County
Phe17.01C05	*Myrmecophilus quadrispina*	Uninfected	*Pheidole* sp.	NA	Taiwan	Renai Township, Nantou County
Phe17.01C06	*Myrmecophilus quadrispina*	Uninfected	*Pheidole* sp.	NA	Taiwan	Renai Township, Nantou County
pheton01.C03	*Myrmecophilus quadrispina*	Uninfected	*Carebara* sp. (*Pheidologeton*)	Uninfected	Taiwan	Shalu Dist., Taichung City
pl349C01	*Myrmecophilus quadrispina*	Uninfected	*Paratrechina longicornis*	*w*LonA	Taiwan	Kinmen
pl349C02	*Myrmecophilus quadrispina*	Uninfected	*Paratrechina longicornis*	*w*LonA	Taiwan	Kinmen
pl367C01	*Myrmecophilus quadrispina*	Uninfected	*Paratrechina longicornis*	*w*LonF	Taiwan	Huisun, Nantou County
pl367C02	*Myrmecophilus quadrispina*	Uninfected	*Paratrechina longicornis*	*w*LonF	Taiwan	Huisun, Nantou County
pl368C01	*Myrmecophilus quadrispina*	Uninfected	*Paratrechina longicornis*	*w*LonF	Taiwan	Renai Township, Nantou County
Cam01C08	*Myrmophilellus pilipes*	*w*Msp6	*Camponotus* sp.	*w*CamA1, *w*CamA2, *w*CamA3	Malaysia	Lebuh Relau, Pulau Pinang
Dia01C02	*Myrmophilellus pilipes*	*w*Msp6	*Diacamma* sp.	Uninfected	Malaysia	Lebuh Relau, Pulau Pinang
mun2-r3-1	*Myrmophilellus pilipes*	*w*Msp6	*Anoplolepis gracilipes*	NA	Malaysia	Pulau Pinang
Plmy100C01	*Myrmophilellus pilipes*	*w*Msp6	*Paratrechina longicornis*	*w*LonF	Malaysia	Air Hitam, Pulau Pinang
Cam01C01	*Myrmophilellus pilipes*	Uninfected	*Camponotus* sp.	*w*CamA1, *w*CamA2, *w*CamA3	Malaysia	Lebuh Relau, Pulau Pinang
Cam01C02	*Myrmophilellus pilipes*	Uninfected	*Camponotus* sp.	*w*CamA1, *w*CamA2, *w*CamA3	Malaysia	Lebuh Relau, Pulau Pinang
Cam01C03	*Myrmophilellus pilipes*	Uninfected	*Camponotus* sp.	*w*CamA1, *w*CamA2, *w*CamA3	Malaysia	Lebuh Relau, Pulau Pinang
Cam01C04	*Myrmophilellus pilipes*	Uninfected	*Camponotus* sp.	*w*CamA1, *w*CamA2, *w*CamA3	Malaysia	Lebuh Relau, Pulau Pinang
Cam01C06	*Myrmophilellus pilipes*	Uninfected	*Camponotus* sp.	*w*CamA1, *w*CamA2, *w*CamA3	Malaysia	Lebuh Relau, Pulau Pinang

Cam01C07	*Myrmophilellus pilipes*	Uninfected	*Camponotus* sp.	*w*CamA1, *w*CamA2, *w*CamA3	Malaysia	Lebuh Relau, Pulau Pinang
Dia01C01	*Myrmophilellus pilipes*	Uninfected	*Diacamma* sp.	Uninfected	Malaysia	Lebuh Relau, Pulau Pinang
mun2-r1-1	*Myrmophilellus pilipes*	Uninfected	*Anoplolepis gracilipes*	NA	Malaysia	Pulau Pinang
mun2-r1-2	*Myrmophilellus pilipes*	Uninfected	*Anoplolepis gracilipes*	NA	Malaysia	Pulau Pinang
mun2-r2-1	*Myrmophilellus pilipes*	Uninfected	*Anoplolepis gracilipes*	NA	Malaysia	Pulau Pinang
mun2-r3-2	*Myrmophilellus pilipes*	Uninfected	*Anoplolepis gracilipes*	NA	Malaysia	Pulau Pinang
mun2-r4-1	*Myrmophilellus pilipes*	Uninfected	*Anoplolepis gracilipes*	NA	Malaysia	Pulau Pinang
mun2-r4-2	*Myrmophilellus pilipes*	Uninfected	*Anoplolepis gracilipes*	NA	Malaysia	Pulau Pinang
mun2-r4-3	*Myrmophilellus pilipes*	Uninfected	*Anoplolepis gracilipes*	NA	Malaysia	Pulau Pinang
plMYBPC01	*Myrmophilellus pilipes*	Uninfected	*Paratrechina longicornis*	NA	Malaysia	Pulau Pinang
plMYUSMC01	*Myrmophilellus pilipes*	Uninfected	*Paratrechina longicornis*	NA	Malaysia	Pulau Pinang
plMYUSMC02	*Myrmophilellus pilipes*	Uninfected	*Paratrechina longicornis*	NA	Malaysia	Pulau Pinang
PlSG04C01	*Myrmophilellus pilipes*	Uninfected	*Paratrechina longicornis*	*w*LonA	Singapore	Mount Faber Park
plmy100C02	*Myrmophilellus pilipes*	Uninfected	*Paratrechina longicornis*	*w*LonF	Malaysia	Air Hitam, Pulau Pinang

Table S4 Primer sequences for *wsp* gene and PCR conditions used in this study

Wolbachia type	Name	Sequences (5'-3')	Ta (°C)	Size (bp)	Reference
Wolbachia universal	81F	TGGTCCAATAAGTGATGAAGAAAC	50	610	[23]
	691R	AAAAATTAAACGCTACTCCA			
*w*Msp4/*w*CamA3	M4F	GGACACAGACATTCATAATCCA	54	308	This study
	M4R	TATAGGTTTGACCATCCACG			
*w*Msp5	M5F	AAAGCTTTTGATCCTTTCA	54	408	This study
	M4R	GCTAGCACCATAAGARCCA			
*w*Mame1	A1F	AAGGTGATAAAGATCAAGATCCTT	54	439	This study
	A1R	TACCATCACCCTTAGTTGTTGCAT			
*w*Mame2	A2F	AGATAATAAAGACCAAGACCT	54	285	This study
	A2R	GGACTCTTTAAAGGATTGCTA			
*w*CamA1/*w*LonA	F	TCCAGCAATTGCAGACAGTT	58	292	This study
	R	GCTTGCTGCAGCAGTATCTTTA			
*w*CamA2	F	TATTACCTATAAGAAAGACAGTAG	54	455	This study
	R	CCTTTGCCGTCTTTGTCAGT			

Figure legends

Figure S1. Alignment of Illumina paired-end sequence reads to the reference MLST sequences (A) *hcpA*, (B) *ftsZ*, (C) *gatB*, (D) *coxA* and (E) *fbpA* of *w*LonF (top), *w*Msp4 (middle), and *w*Msp5 (bottom). Note only those reads perfectly matching the reference sequences were shown.

Figure S2 Genealogical relationships of *Wolbachia* strains. (a) Maximum Likelihood (ML) tree and (b) subtrees for *Wolbachia* strains based on *wsp* gene. Black circles indicate sequences data generated in this study. Strains are represented by the infected arthropod host species with which they are associated. *Wolbachia* from ant crickets, ants, and orthoptera are colored yellow, blue, and pink respectively.

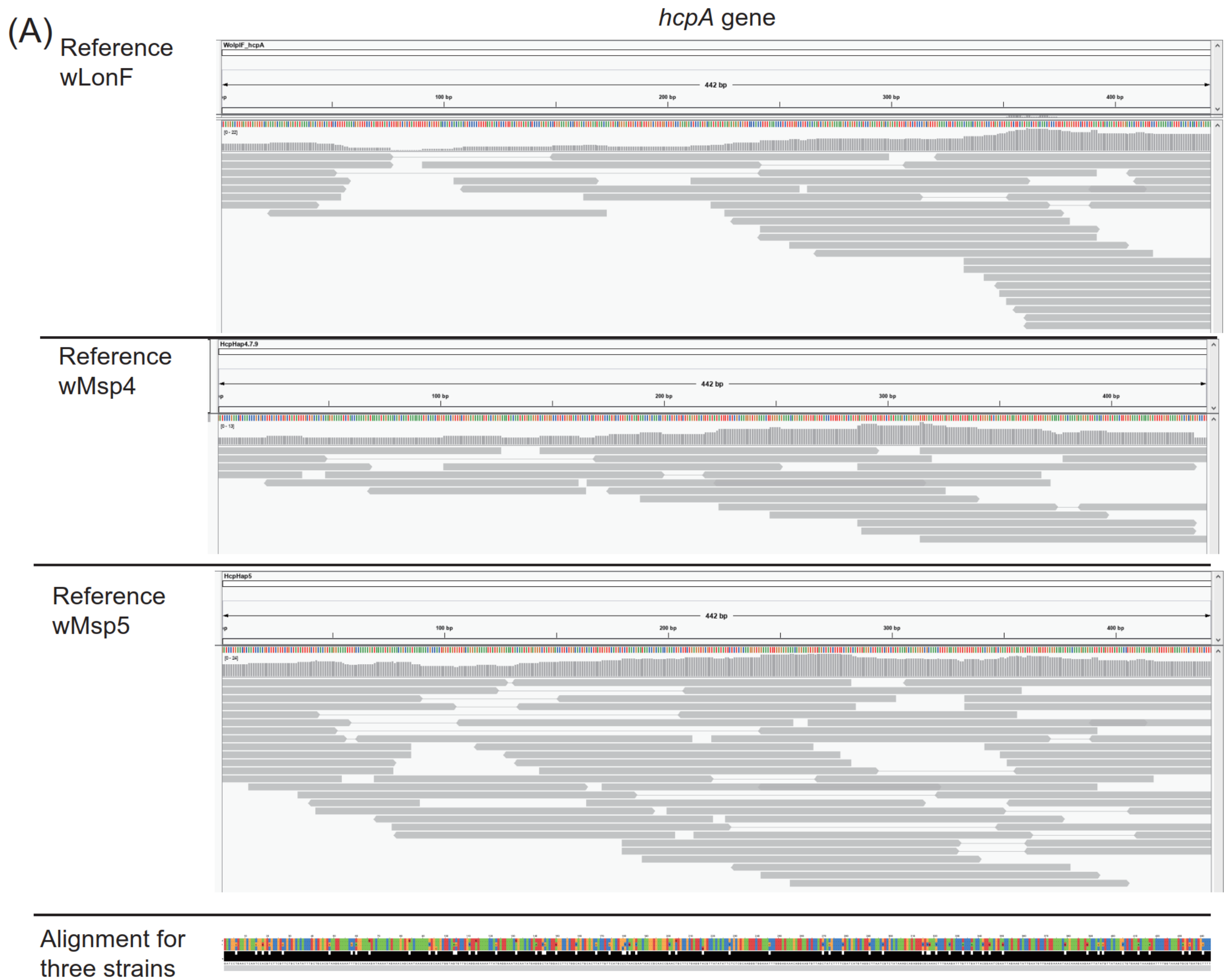
(A)
hcpA gene
Reference wLonF
WolpIF_hcpA
442 bp
100 bp
200 bp
300 bp
400 bp
Reference wMsp4
HcpHap4.7.9
Reference wMsp5
HcpHap5
Alignment for three strains

Figure S1

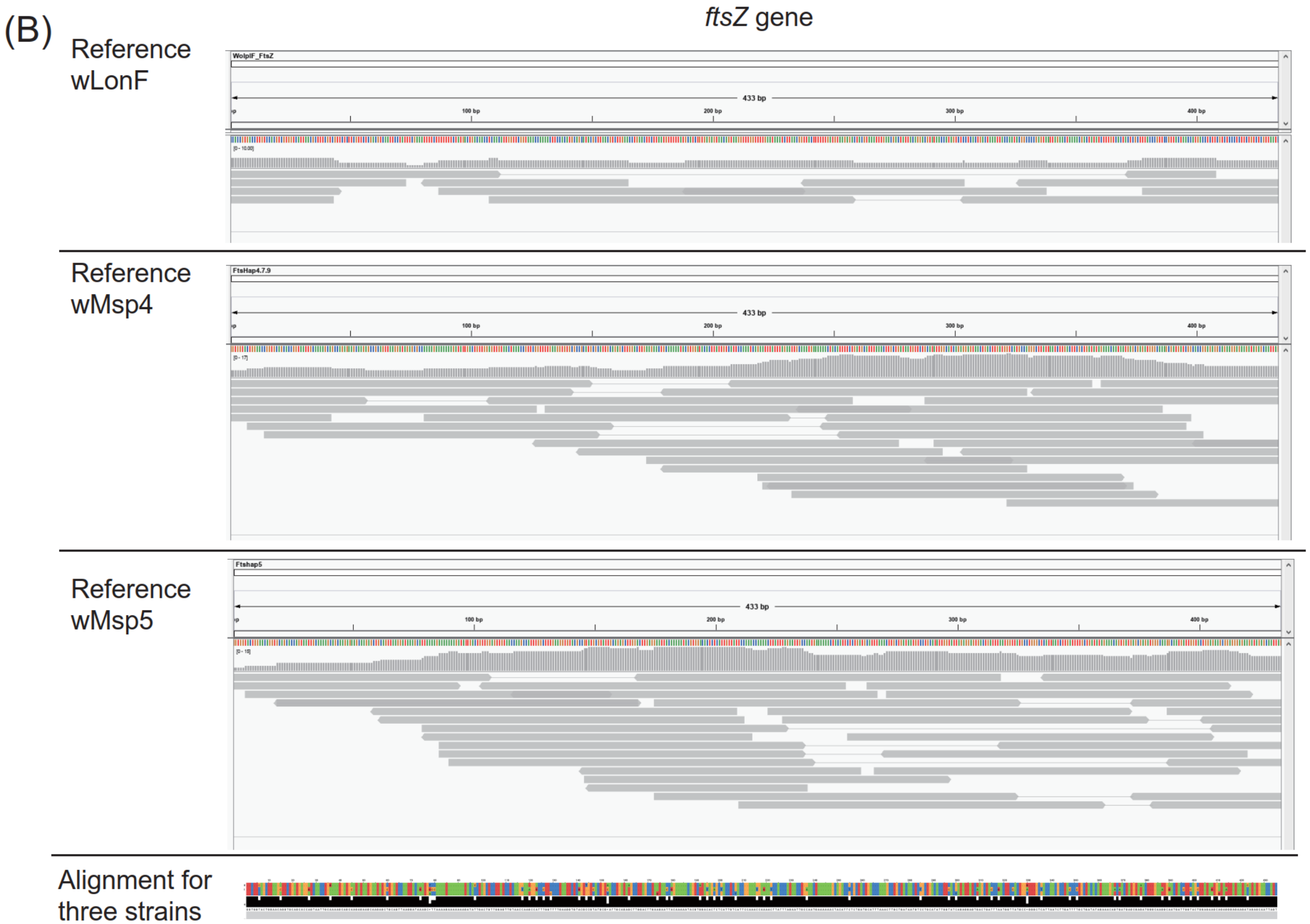

Figure S1 (Continued)

(C)

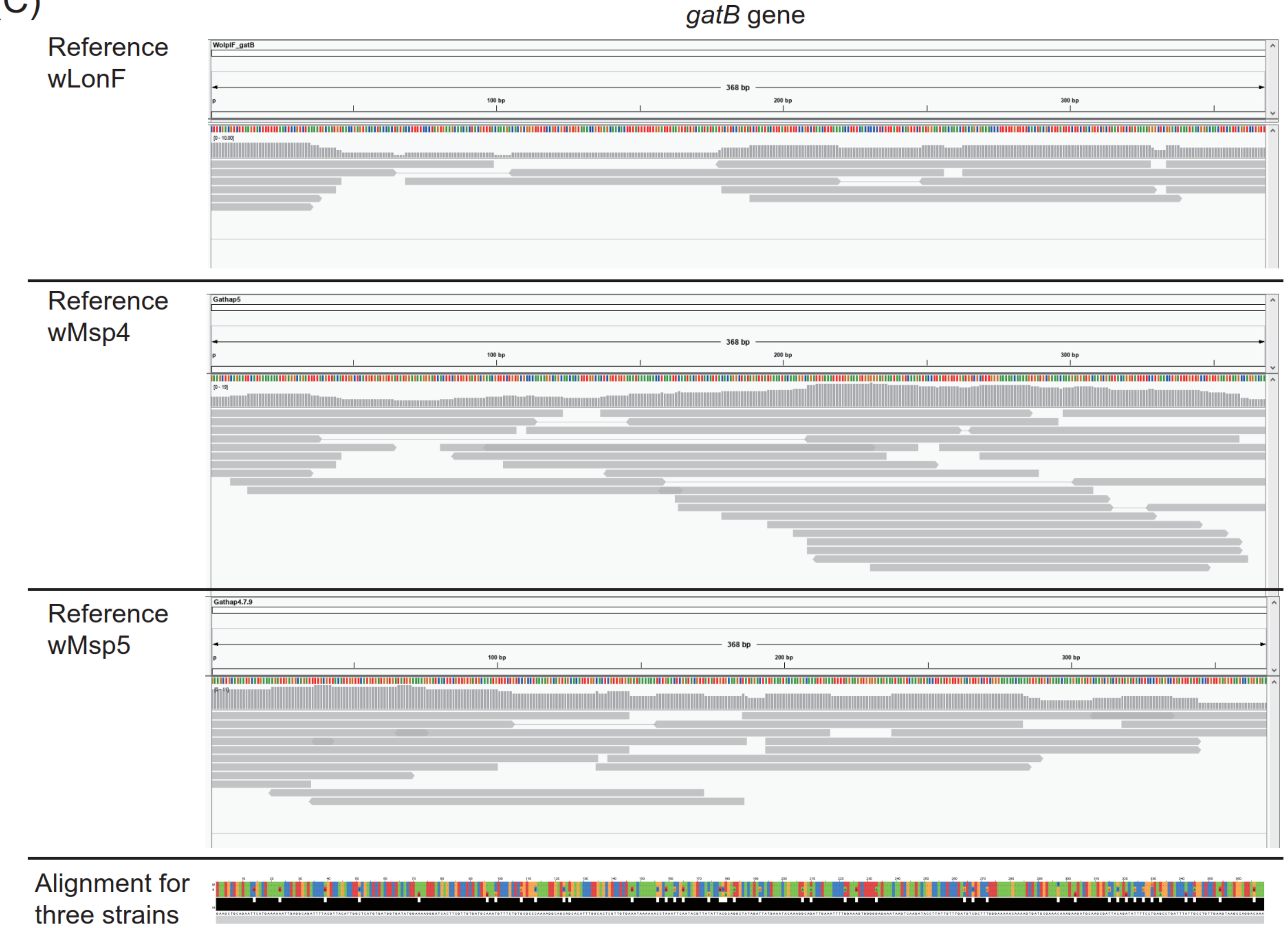

Figure S1 (Continued)

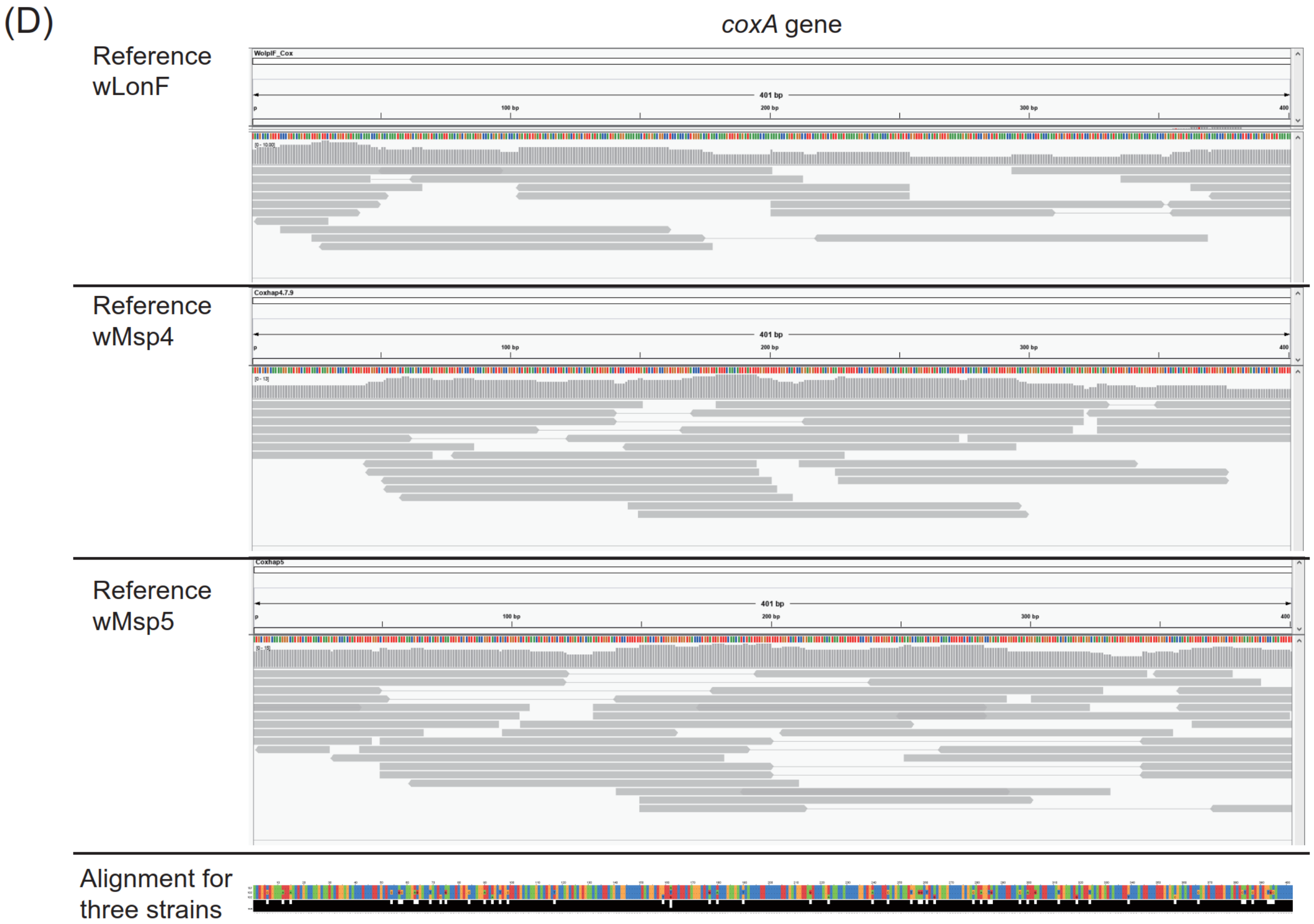

Figure S1 (Continued)

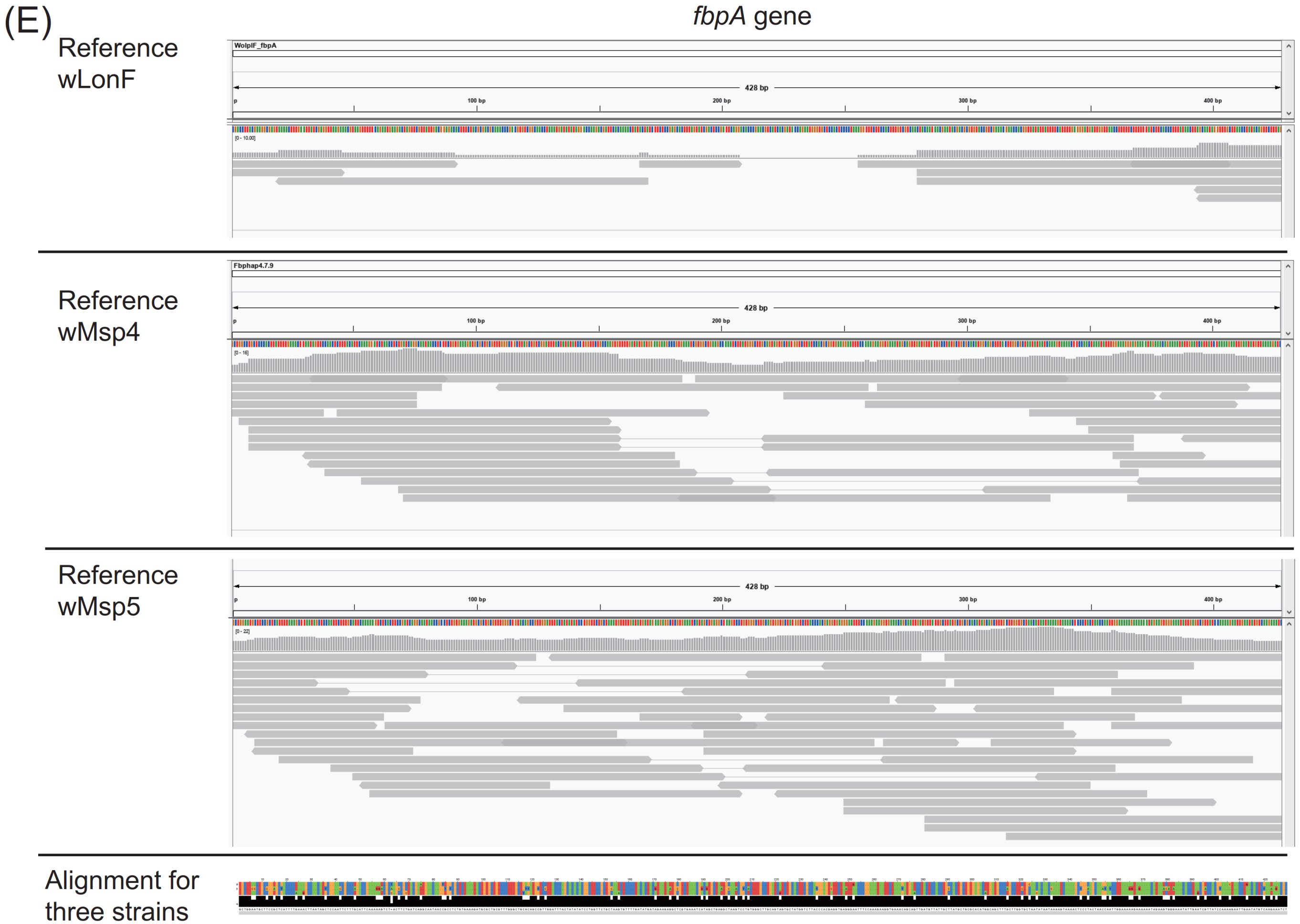

Figure S1 (Continued)

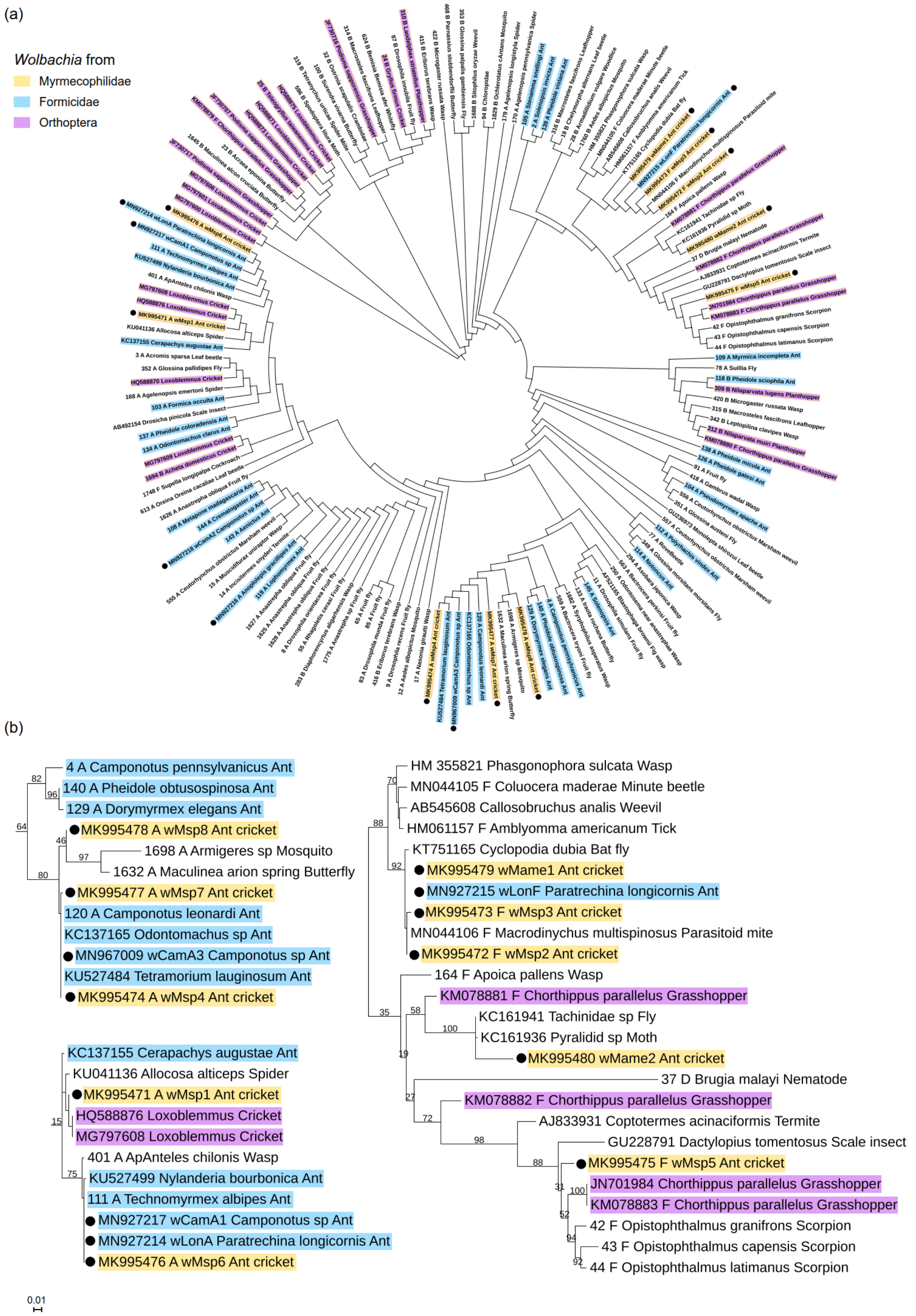
(a)
Wolbachia from
Myrmecophilidae
Formicidae
Orthoptera
(b)
4 A Camponotus pennsylvanicus Ant
140 A Pheidole obtusospinosa Ant
129 A Dorymyrmex elegans Ant
MK995478 A wMsp8 Ant cricket
1698 A Armigeres sp Mosquito
1632 A Maculinea arion spring Butterfly
MK995477 A wMsp7 Ant cricket
120 A Camponotus leonardi Ant
KC137165 Odontomachus sp Ant
MN967009 wCamA3 Camponotus sp Ant
KU527484 Tetramorium lauginosum Ant
MK995474 A wMsp4 Ant cricket
KC137155 Cerapachys augustae Ant
KU041136 Allocosa alticeps Spider
MK995471 A wMsp1 Ant cricket
HQ588876 Loxoblemmus Cricket
MG797608 Loxoblemmus Cricket
401 A ApAnteles chilonis Wasp
KU527499 Nylanderia bourbonica Ant
111 A Technomyrmex albipes Ant
MN927217 wCamA1 Camponotus sp Ant
MN927214 wLonA Paratrechina longicornis Ant
MK995476 A wMsp6 Ant cricket
0.01
HM 355821 Phasgonophora sulcata Wasp
MN044105 F Coluocera maderae Minute beetle
AB545608 Callosobruchus analis Weevil
HM061157 F Amblyomma americanum Tick
KT751165 Cyclopodia dubia Bat fly
MK995479 wMame1 Ant cricket
MN927215 wLonF Paratrechina longicornis Ant
MK995473 F wMsp3 Ant cricket
MN044106 F Macrodinychus multispinosus Parasitoid mite
MK995472 F wMsp2 Ant cricket
164 F Apoica pallens Wasp
KM078881 F Chorthippus parallelus Grasshopper
KC161941 Tachinidae sp Fly
KC161936 Pyralidid sp Moth
MK995480 wMame2 Ant cricket
37 D Brugia malayi Nematode
KM078882 F Chorthippus parallelus Grasshopper
AJ833931 Coptotermes acinaciformis Termite
GU228791 Dactylopius tomentosus Scale insect
MK995475 F wMsp5 Ant cricket
JN701984 Chorthippus parallelus Grasshopper
KM078883 F Chorthippus parallelus Grasshopper
42 F Opistophthalmus granifrons Scorpion
43 F Opistophthalmus capensis Scorpion
44 F Opistophthalmus latimanus Scorpion

Figure S2
